# Coordination Chemistry and Methylation of Mixed‐Substituted Tetraphosphetanes (RP−P*t*Bu)_2_ (R=Ph, Py)

**DOI:** 10.1002/chem.202001360

**Published:** 2020-08-17

**Authors:** Robin Schoemaker, Philipp Kossatz, Kai Schwedtmann, Felix Hennersdorf, Jan J. Weigand

**Affiliations:** ^1^ Faculty of Chemistry and Food Chemistry Technische Universität Dresden 01062 Dresden Germany

**Keywords:** cations, coinage metal complexes, phosphorus, tetraphosphetanes

## Abstract

Synthesis of mixed‐substituted tetraphosphetanes (RP−P*t*Bu)_2_ (R=Ph (**4**), Py (**5**); Py=2‐pyridyl) is achieved from the condensation of dipyrazolylphosphanes RPpyr_2_ (R=Py (**1**), Ph (**3**); pyr=3,5‐dimethylpyrazolyl) as P_1_‐building block (R−P) and *t*BuPH_2_ in an equimolar ratio. Compound **5** is of special interest since the presence of two pyridyl‐substituents as well as the P_4_‐core allows for a rich coordination chemistry with coinage metal salts [Cu(MeCN)_4_][OTf], Ag[OTf] and in situ formed [Au(tht)][OTf] (tht=tetrahydrothiophene). Both tetraphosphetanes undergo alkylation reaction with MeOTf to give a series of tetraphosphetanium and tetraphosphetanediium triflate salts with additional methylation of the pyridyl‐moiety in case of **5** resulting in interesting novel cyclic trications. Harsh reaction condition and an excess of MeOTf converts **5** into the cyclic trication [‐P(^Me^Py)PMe_2_P(^Me^Py)P*t*Bu‐]^3+^ (**13** ^3+^; ^Me^Py=1‐methylpyridiniumyl) through the elimination of *iso*butene. This salt undergoes a complicated rearrangement reaction involving a P−P/P−P bond metathesis to form trication [‐P(MePy)_3_P*t*Bu‐]^3+^ (**17** ^3+^) when reacted with Me_2_PPMe_2_.

## Graphical abstract

**Choose your donor**: A convenient route towards mixed‐substituted tetraphosphetanes was established, enabling the synthesis of (PhP‐P*t*Bu)_2_ and (PyP‐P*t*Bu)_2_ (Py=2‐pyridyl) in good yields. The coordination chemistry of (PyP‐P*t*Bu)_2_ was investigated by reacting it with coinage metal triflate salts. Further methylation studies on both tetraphosphetanes revealed an interesting chemistry of multiply charged, cylic polyphosphanes.

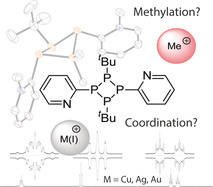

## Introduction

Cyclic polyphosphanes are a well‐known substance class in poly‐phosphorus chemistry.^1^ Most commonly, these compounds are synthesized by condensation^2^ or reduction^3^ of corresponding dihalophosphanes RPX_2_ (X=halogen) yielding symmetrical, mono‐substituted derivatives of type (RP)_*n*_ (*n*=3–6), which undergo rearrangement to the respective thermodynamically favored ring‐sizes (typically tetraphosphetanes or pentaphospholanes, which are also known as cyclotetraphosphanes and cyclopentaphosphanes, respectively; both nomenclatures are equivalent).1a This sort of scrambling reaction strongly depends on the substituents and the polarity of the used solvents.^1^, ^4^ Dihalophosphanes featuring sterically demanding substituents give access to diphosphenes RP=PR,^5^ which under certain circumstances dimerize to the respective tetraphosphetanes (RP)_4_.^6^ However, classical routes for the formation of mixed‐substituted tetraphosphetanes remain very often unselective, thus, there are only a few reports on mixed‐substituted tetraphosphetanes using specialized synthetic protocols.^6^, ^7^, ^8^ Noteworthy, reacting the sterically encumbered phosphane Mes*PH_2_ (Mes*=2,4,6‐tri‐*tert*‐butylphenyl) with PCl_3_ in the presence of a base, Schulz and co‐workers succeeded in the synthesis of the dichloro‐substituted tetraphosphetane (Mes*P‐PCl)_2_,8a showing a remarkable follow‐up chemistry, such as the reduction to bicyclic tetraphosphane Mes*P_4_Mes*.^8^ However, a more general route would certainly be beneficial for further exploration of the chemistry of this type of compounds.

**Figure 1:**
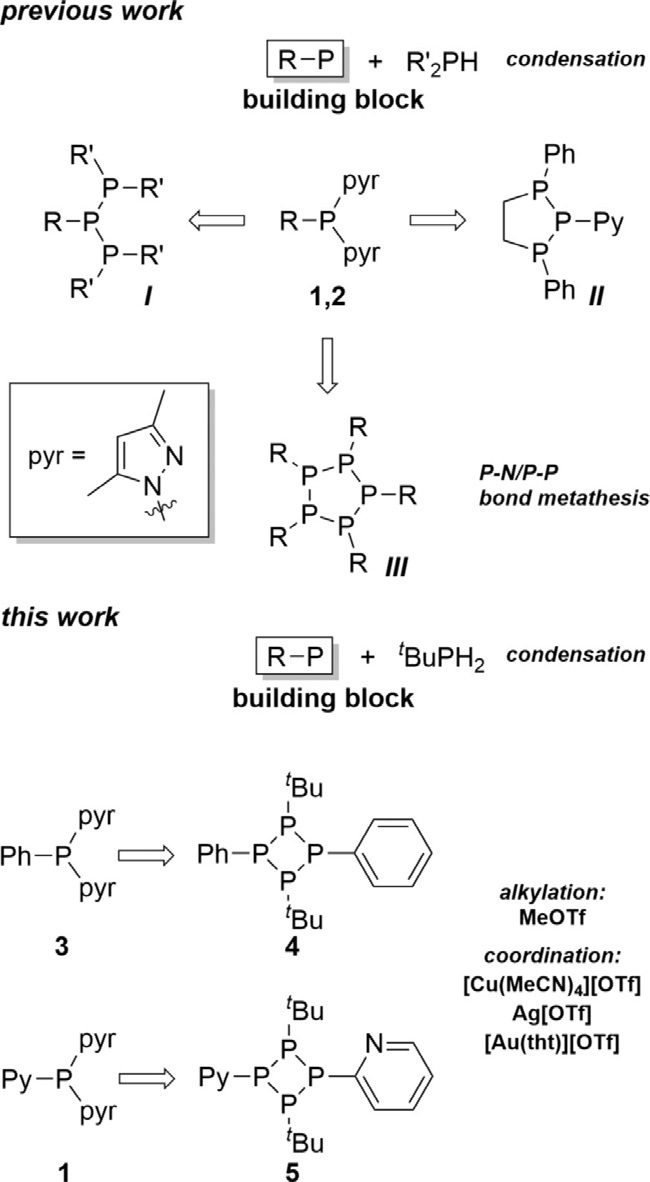
Previous work: Synthesis of triphosphanes (***I***), triphospholane (***II***) and pentaphospholanes (***III***) from dipyrazolylphosphanes RPPyr_2_.^3^ This work: Synthesis of mixed‐substituted tetraphosphetanes **4** und **5** from RPpyr_2_ and *t*BuPH_2_ and selected follow‐up chemistry.

As part of our ongoing development of methodologies using pyrazolyl‐substituted phosphanes as P_1_‐building blocks in P−P bond forming reactions by condensation or P−N/P−P bond metathesis,^9^, ^10^ we recently reported on the targeted synthesis of a series of polyphosphorus compounds including acyclic and cyclic polyphosphanes (Figure 1).^11^ The controlled reactions of dipyrazolylphosphanes RPpyr_2_ **1** and **2** (pyr=3,5‐dimethylpyrazolyl; R=Py (**1**), BTz (**2**); Py=2‐pyridyl, BTz=2‐benzothiazolyl) with secondary phosphanes (R’_2_PH; PhPH(CH_2_)_2_PHPh; R’=Cy, *t*Bu; Figure 1) yields triphosphanes (***I***) and a triphospholane (***II***) by condensation or pentaphospholanes (***III***) through a P−N/P−P bond metathesis reaction.^11^ Our protocol allows for the synthesis of these compounds on a multigram scale and, thus, offers their use as multidentate ligands in coordination chemistry as well as follow‐up chemistry, such as alkylation reactions. Extending this protocol towards primary phosphanes (RPH_2_) should generally allow the formation of cyclophosphanes. Thus, we selected *t*BuPH_2_ as sterically demanding primary phosphane and reacted it with dipyrazolylphosphanes RPpyr_2_ (R=Py (**1**), Ph (**3**)) as P_1_‐building block (R−P) in a 1:1 ratio in order to selectively access tetraphosphetanes **4** and **5** (Figure 1).

**Figure 2:**
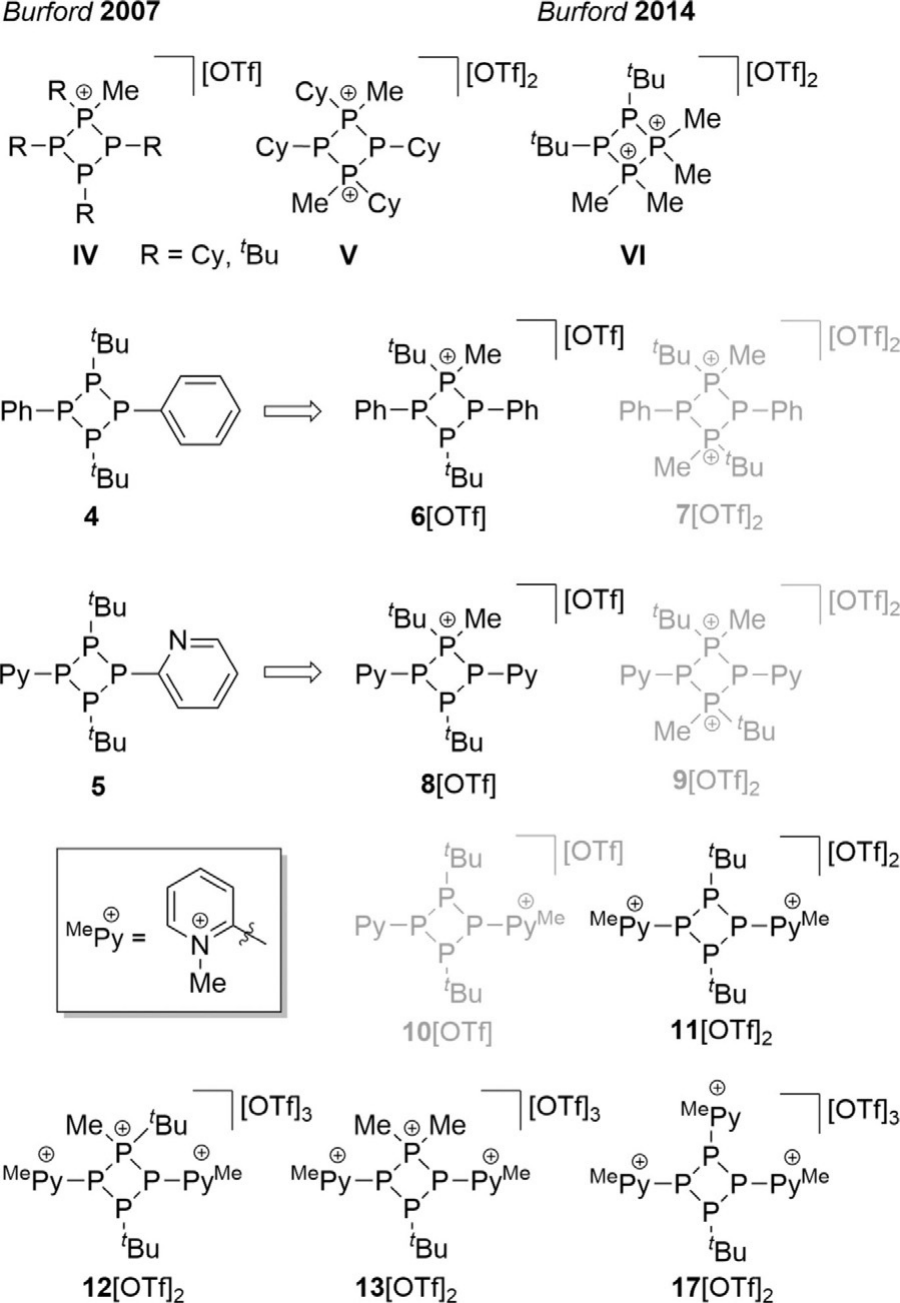
Several triflate salts derived from tetraphosphetanes **4** and **5** compared to established salts **IV**–**VI**.^12^

Particularly, compound **5** is of special interest since the presence of two pyridyl‐substituents as well as the P_4_‐core should allow for a rich coordination chemistry which we probed in the reaction with the coinage metal salts [Cu(MeCN)_4_][OTf], Ag[OTf] and in situ prepared [Au(tht)][OTf] (tht=tetrahydrothiophene). Both tetraphosphetanes were excessively alkylated with MeOTf in order to investigate their capability in the formation of multiply charged cations, related to those reported by Burford and co‐workers (**IV**‐**VI**, Figure 2).^12^ Equimolar reactions of **4** or **5** with MeOTf yield the mono‐methylated salts **6**[OTf] and **8**[OTf], whereas the formation of dicationic salts **7**[OTf]_2_ and **9**[OTf]_2_, similar to **V**, are not observed if the amount of MeOTf is increased. The presence of the pyridyl‐substituents in **5** offers an additional alkylation site, thus, multiply charged cations are accessible. Increasing the amount of MeOTf, we observe a preference for the alkylation of pyridyl‐substituents over the P_4_‐core. However, mono‐methylation to salt **10**[OTf] is not observed since the formation of a mixture of di‐methylated **11**[OTf]_2_ and trimethylated **12**[OTf]_3_ is preferred. Harsh reaction condition and a large excess of MeOTf converts **5** into tricationic salt **13**[OTf]_3_ which undergoes a complicated rearrangement reaction to salt **17**[OTf]_3_ when reacted with Me_2_PPMe_2_, by means of a P−P/P−P bond metathesis reaction. A detailed discussion of our findings is presented in the following.

## Results and Discussion

**Scheme 1:**
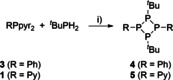
Preparation of tetraphosphetanes **4** and **5**; i) −2 pyrH, MeCN, −30 °C, 16 h, 69 % (**4**), 53 % (**5**).

The mixed‐substituted tetraphosphetanes **4** (R=Ph) and **5** (R=Py) form readily from the reaction of *t*BuPH_2_ and the respective dipyrazolylphosphanes RPpyr_2_ (R=Py (**1**), Ph (**3**)) when mixed in equimolar ratios in CH_3_CN at −30 °C (Scheme 1).^13^

After evaporating the solvent and subsequent sublimation of the by‐product 3,5‐dimethylpyrazole, both compounds are obtained as colorless powders in quantitative yields and an acceptable purity of >90 %, deduced from the integral ratios in the ^31^P NMR spectra.^14^ Washing of the crude products with cold MeCN gives analytically pure materials, however, reduces the isolated yield significantly (**4**: 69 %, **5**: 53 %).^14^

**Figure 3:**
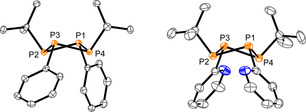
Molecular structures of tetraphosphetanes **4** (left) and **5** (right) (hydrogen atoms are omitted for clarity; thermal ellipsoids are displayed at 50 % probability); selected bond lengths and angles are given in Table 3.

**Scheme 2:**
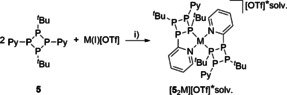
Reaction of **5** with selected coinage metal triflate salts “M^I^[OTf]”; i) CH_2_Cl_2_, r.t., 1 h; M=Cu, M[OTf]=[Cu(MeCN)_4_][OTf], −4 MeCN, 89 %; M=Ag, M[OTf]=Ag[OTf], 76 %; M=Au, M[OTf]=(tht)AuCl+Me_3_SiOTf, ‐Me_3_SiCl, ‐tht, 74 %.

**Figure 4:**
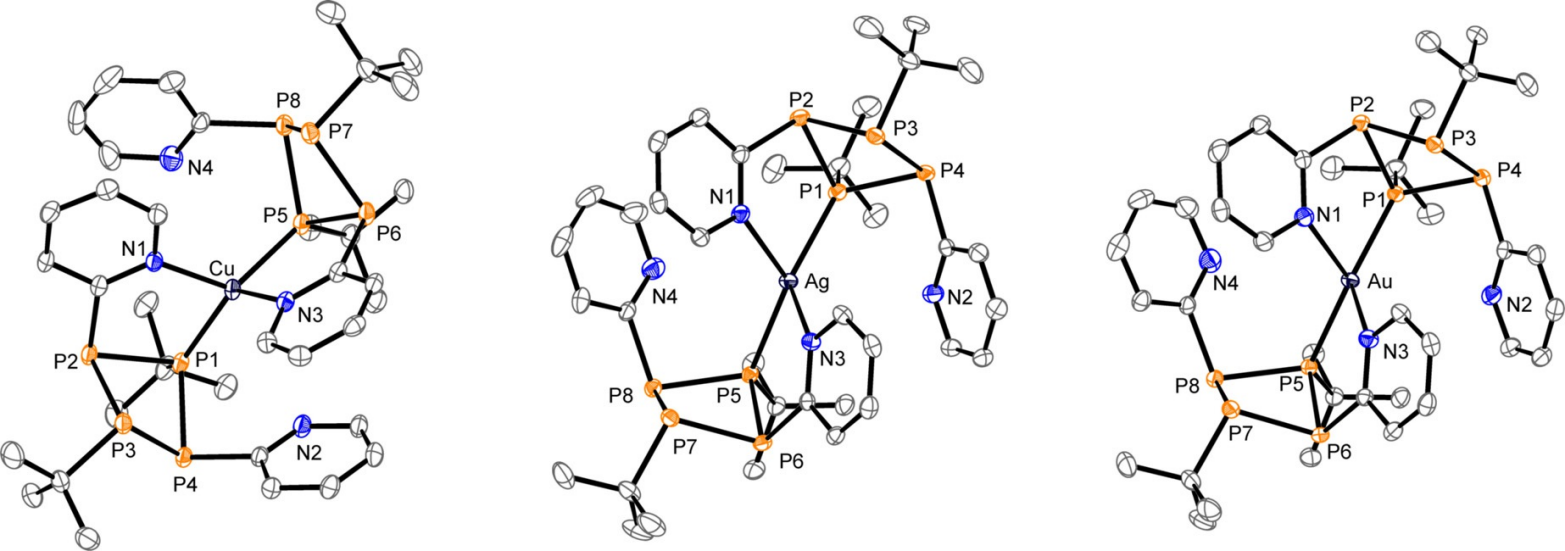
Molecular structures of [Cu**5**_2_]^+^ in [Cu**5**_2_][OTf]*CH_2_Cl_2_ (left), [Ag**5**_2_]^+^ in [Ag**5**_2_][OTf]*MeCN (middle) and [Au**5**_2_]^+^ in [Au**5**_2_][OTf]*CH_2_Cl_2_ (right) (hydrogen atoms, anions and solvate molecules are omitted for clarity, thermal ellipsoids are displayed at 50 % probability); selected bond lengths (Å) and angles (°) are given in Table 1.

Notably, the formation of other ring sizes is not observed and for both compounds the observation of an A_2_X_2_ spin system in the ^31^P NMR spectrum [**4**: *δ*(P_A_)=−88.4 ppm, *δ*(P_X_)=−15.5 ppm; ^1^ *J*(P_A_P_X_)=−130 Hz; **5**: *δ*(P_A_)=−81.9 ppm, *δ*(P_X_)=−18.8 ppm; ^1^ *J*(P_A_P_X_)=−131 Hz] confirms the *C*_2*v*_ symmetry of the molecules. The structural connectivity is moreover confirmed by single‐crystal X‐ray analysis and the molecular structures of **4** and **5** are depicted in Figure 3. The structural parameters of the P_4_‐cores of **4** and **5** are listed in Table 3 and compare well with known symmetrically, homosubstituted tetraphosphetanes such as (PhP)_4_ 15 and (CyP)_4_ 16 which made us abstain from a detailed discussion. While the coordination behavior of tetraphosphetanes of type (RP)_4_ (R=Me, Et, Ph) has been extensively explored,^17^ corresponding investigations with mixed‐substituted derivatives are scarce and, to the best of our knowledge, did not involve derivatives with pyridyl‐substituents. The additional nitrogen‐based donor site of the pyridiyl‐units in **5** makes it a suitable multidentate ligand for metal coordination with selected Cu^I^, Ag^I^ and Au^I^ triflate salts. Thus, we reacted **5** in a 2:1 ratio in CH_2_Cl_2_ with [Cu(MeCN)_4_][OTf] and Ag[OTf] and in the case of Au^I^, with the corresponding triflate salt which was in situ prepared from (tht)AuCl (tht=tetrahydrothiophene) and Me_3_SiOTf (Scheme 2). Vapor diffusion of *n*‐pentane into the reaction mixtures at −30 °C yields crystals of [Cu**5**_2_][OTf]*CH_2_Cl_2_, [Ag**5**_2_][OTf]*CH_2_Cl_2_ and [Au**5**_2_][OTf]*CH_2_Cl_2_ in good to very good yields (74–89 %). Crystals of [Cu**5**_2_][OTf]*CH_2_Cl_2_ and [Au**5**_2_][OTf]*CH_2_Cl_2_ are suitable for X‐ray analysis, however, better quality crystals of [Ag**5**_2_][OTf] are obtained as MeCN monosolvate by recrystallization from MeCN/Et_2_O. The molecular structures are depicted in Figure 4 and structural parameters are summarized in Table 1. In all three cases the molecular structures reveal mononuclear metal complexes where the metal center M^I^ is coordinated by two tetraphosphetanes through one *t*BuP‐moiety and the N atom of one pyridyl‐substituent each. The P−P bond lengths in the complexes remain virtually unchanged compared to the free tetraphosphetane **5**. The average P−M distances in [M**5**_2_][OTf] [2.2621 Å (Cu), 2.4100 Å (Ag) and 2.2982 Å (Au)] are comparable to those reported for other coinage metal complexes of polyphosphanes.^11^ [Cu**5**_2_]^+^ shows a distorted tetrahedral geometry around the copper atom with a N‐Cu‐N angle of 94.55(6)° and a P‐Cu‐P angle of 127.26(2)°. This distortion causes a slight elongation of the Cu−N bond lengths in [Cu**5**_2_]^+^ (av. 2.1124 Å) compared to tetrakis(pyridine)copper(I) hexafluorophosphate (Cu−N 2.061(3) Å), which shows an almost perfect tetrahedral coordination geometry.^18^

**Table 1:** Selected geometrical parameters of crystallographically characterized coinage metal complexes [Cu**5**_2_][OTf]⋅CH_2_Cl_2_, [Ag**5**_2_][OTf]⋅MeCN and [Au**5**_2_][OTf]⋅CH_2_Cl_2_.

	[Cu**5**_2_][OTf]	[Ag**5**_2_][OTf]	[Au**5**_2_][OTf]
P‐Pa in Å	2.2204	2.2183	2.2191
P‐M^a^ in Å	2.2621	2.4100	2.2982
*N*‐M^a^ in Å	2.1124	2.4458	2.6262
*N*‐M‐N in °	94.55(6)	85.18(5)	75.42(6)
P‐M‐P in °	127.26(2)	141.14(2)	154.71(2)

[a] average bond lengths and angles are given.

**Figure 5:**
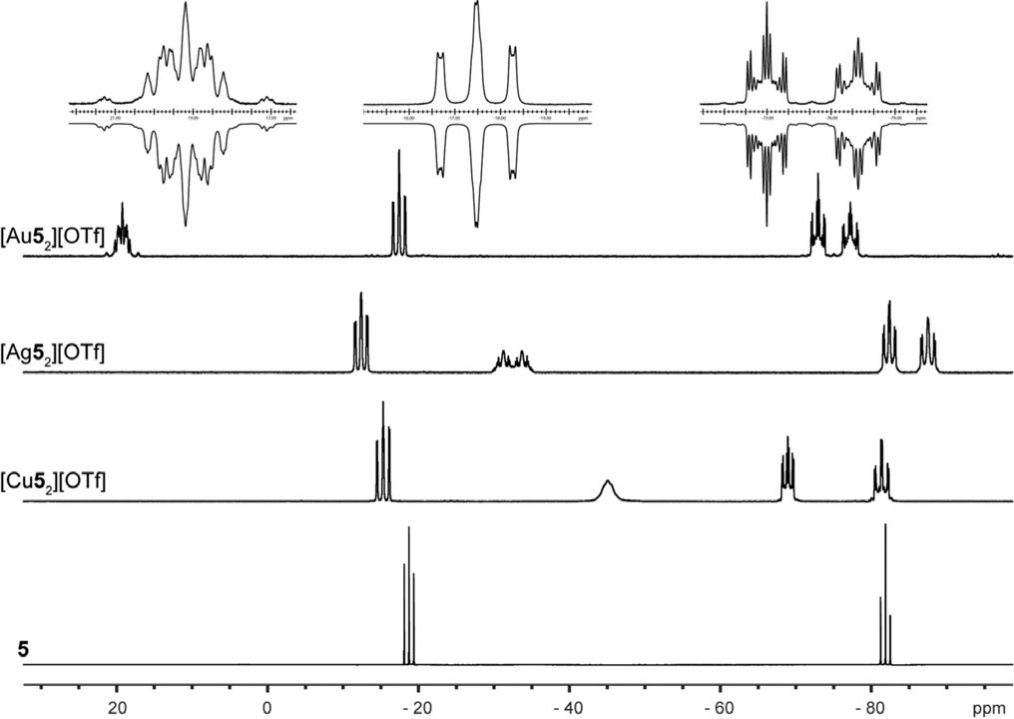
^31^P{^1^H} spectra of **5** (CD_2_Cl_2_, 300 K) and [Cu**5**_2_][OTf], [Ag**5**_2_][OTf] and [Au**5**_2_][OTf] (CD_2_Cl_2_, 190 K); insets show the AA'BB'MM'XX’ spin system of the experimental (upwards) and fitted spectra (downwards) of [Au**5**_2_][OTf].

Cations [Ag**5**_2_]^+^ and [Au**5**_2_]^+^ show wider P‐M‐P angles ([Ag**5**_2_]^+^: 141.14(2)° and [Au**5**_2_]^+^ 154.71(2)°) and more acute N‐M‐N angles ([Ag**5**_2_]^+^: 85.18(5)° and [Au**5**_2_]^+^ 75.42(6)°), causing a further elongation of the N−M distances ([Ag**5**_2_]^+^: 2.4458 Å and [Au**5**_2_]^+^ 2.6262 Å (average values). This states a decreasing participation of the pyridine nitrogen and vice versa an increasing involvement of the phosphorus in the coordination of silver and gold, which is in consistency with Pearsons’ concept.^19^ We further analyzed coordination complexes [Cu**5**_2_][OTf], [Ag**5**_2_][OTf] and [Au**5**_2_][OTf] by multinuclear NMR spectroscopy at various temperatures. All three complexes [Cu**5**_2_][OTf], [Ag**5**_2_][OTf] and [Au**5**_2_][OTf] show a dynamic behavior in CD_2_Cl_2_ solution at 290 K. In the ^1^H NMR spectra four resonances for the pyridyl moieties are observed in each case of the three coordination complexes, stating the fast exchange of the pyridyl moieties coordinating the respective metal cation. Upon cooling this exchange is slowing down, leading to eight different resonances for the pyridyl moieties. While two pyridyl groups are coordinating the metal cation, the other two are not, making them chemically inequivalent. The ^1^H NMR spectra of [Cu**5**_2_][OTf], [Ag**5**_2_][OTf] and [Au**5**_2_][OTf] at temperatures from 290‐190 K are depicted in the supporting information,^20^ showing coalescence temperatures of around 260 K for [Cu**5**_2_][OTf] and of 210 K for [Ag**5**_2_][OTf] and [Au**5**_2_][OTf]. These findings are also observed in the ^31^P NMR spectra.^20^ [Au**5**_2_][OTf] and [Cu**5**_2_][OTf] show three broadened resonances at 300 K due to dynamic processes and additionally for [Cu**5**_2_][OTf] due to the fast quadrupole relaxation of the ^63^Cu nucleus.^21^ For [Ag**5**_2_][OTf] two resonances are observed at 300 K. Measuring the ^31^P NMR spectra at 190 K reveals an AA'BB'MM'XX’ spin system for each coinage metal complex [M**5**_2_][OTf] (see Figure 5; Table 2). Details on the coupling constants for [Au**5**_2_][OTf], acquired by iteratively fitting the spectrum, are reported in the Supporting Information. Severe line broadening in the spectra of [Cu**5**_2_][OTf] due to the fast quadrupole relaxation of the ^63^Cu nuclei,^21^ and further line splitting in the spectra of [Ag**5**_2_][OTf] as a result of the complexation with the ^107^Ag/^109^Ag nuclei, made us refrain from iteratively fitting these spectra. It is noteworthy that the resonance of the *t*Bu−P moiety coordinating the metal cation shows a significant shift compared to the *t*Bu−P moiety of the free ligand **5**. Coordination to Cu^I^ and Ag^I^ cause upfield shifts of Δ*δ*=26.3 ppm and Δ*δ*=13.7 ppm, respectively. Yet coordination to Au^I^ is observed by a downfield shift of Δ*δ*=38.0 ppm (Figure 5; Table 2).

**Table 2:** Chemical shifts of **5**, [Cu**5**_2_][OTf]*CH_2_Cl_2_, [Ag**5**_2_][OTf]*MeCN and [Au**5**_2_][OTf]*CH_2_Cl_2_ in ppm; entries in blue indicate resonances of the *t*Bu‐P moiety coordinating the metal(I) cation.

	**5**	[Cu**5**_2_][OTf]	[Ag**5**_2_][OTf]	[Au**5**_2_][OTf]
*δ*(P_A_)	−81.9	−81.4	−87.6	−77.3
*δ*(P_B_)	–	−68.9	−82.4	−73.0
*δ*(P_M_)	–	−45.1	−32.5	−17.5
*δ*(P_X_)	−18.8	−15.4	−12.4	19.2

**Scheme 3:**
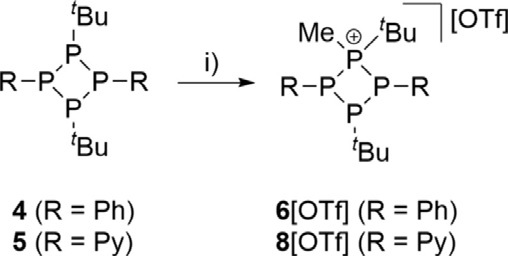
Mono‐methylation reaction of **4** and **5**; i)+MeOTf, Et_2_O, r.t., 16 h, 87 % (**6**[OTf]), 91 % (**8**[OTf]).

**Figure 6:**
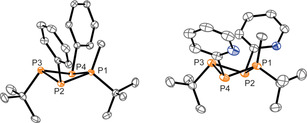
Molecular structures of **6**[OTf] (left) and **8**[OTf] (right) (hydrogen atoms and anions are omitted for clarity; thermal ellipsoids are displayed at 50 % probability); selected bond lengths and angles are given in Table 3.

We further investigated the donor ability of compounds **4** and **5** by methylation reactions with an equimolar ratio of MeOTf in Et_2_O (Scheme 3). For both compounds, colorless precipitates are obtained which after filtration and subsequent recrystallization from MeCN/Et_2_O were identified as tetraphosphetane‐1‐ium triflate salts **6**[OTf] and **8**[OTf]. Both salts are obtained in very good yield (**6**[OTf]: 87 %; **8**[OTf]: 91 %) and their ^31^P{^1^H} NMR spectra display an A_2_MX spin system each [**6**[OTf]: *δ*(P_A_)=−81.2 ppm, *δ*(P_M_)=−39.8 ppm, *δ*(P_X_)=22.0 ppm; ^1^ *J*(P_A_P_X_)=−248 Hz, ^1^ *J*(P_A_P_M_)=−127 Hz, ^2^ *J*(P_M_P_X_)=23 Hz; **8**[OTf]: *δ*(P_A_)=−70.5 ppm, *δ*(P_M_)=−24.9 ppm, *δ*(P_X_)=24.2 ppm; ^1^ *J*(P_A_P_X_)=−225 Hz, ^1^ *J*(P_A_P_M_)=−132 Hz, ^2^ *J*(P_M_P_X_)=15 Hz] as expected for the mono‐methylation of one of the *t*Bu−P moieties. X‐ray analysis of both salts confirmed our findings and the molecular structures of cations **6** ^+^ and **8** ^+^ are shown in Figure 6 and their structural parameters are in good agreement with those reported for related mono‐methylated tetraphosphetanium cations (Table 3).12b, 12c

**Table 3:** Selected geometrical parameters of crystallographically characterized 4, 5, **6**[OTf], **8**[OTf], **11**[OTf]_2_, **12**[OTf]_3_⋅2 MeNO_2_, **13**[OTf]⋅3 MeNO_2_ and **17**[OTf]; bond lengths are given in Å, angles are given in °; entries in blue indicate λ^3^P−λ^4^P^+^ bond lengths and λ^3^P‐λ^4^P^+^‐λ^3^P bond angles.

	**4**	**5**	**6**[OTf]	**8**[OTf]	**11**[OTf]_2_	**12**[OTf]_3_	**13**[OTf]_3_	**17**[OTf]_3_
P1−P2	2.2236(4)	2.2226(4)	2.1983(6)	2.1899(6)	2.2187(5)	2.2034(5)	2.247(2)	2.2398(7)
P2−P3	2.2222(4)	2.2232(5)	2.2341(6)	2.2419(6)	2.2345(5)^[a]^	2.2120(5)	2.238(2)	2.2194(7)
P3−P4	2.2198(4)	2.2195(4)	2.2333(5)	2.2397(6)	–	2.2420(5)	2.203(2)	2.2224(7)
P4−P1	2.2313(4)	2.2191(4)	2.1938(6)	2.1861(6)	–	2.2351(5)	2.204(2)	2.2257(7)
P1‐P2‐P3	87.87(1)	85.55(2)	83.97(2)	82.46(2)	86.30(2)^[a]^	90.33(2)	83.61(6)	82.92(3)
P2‐P3‐P4	85.94(1)	85.14(2)	90.28(2)	85.89(2)	87.21(2)^[a]^	84.12(2)	81.51(6)	84.44(3)
P3‐P4‐P1	87.73(1)	85.72(2)	84.09(2)	82.59(2)	–	88.76(2)	85.44(6)	83.17(9)
P4‐P1‐P2	85.63(1)	85.16(2)	92.77(2)	88.49(2)	–	84.48(2)	81.29(5)	83.88(2)

[a] P1’≙P3, P2’≙P4.

**Figure 7:**
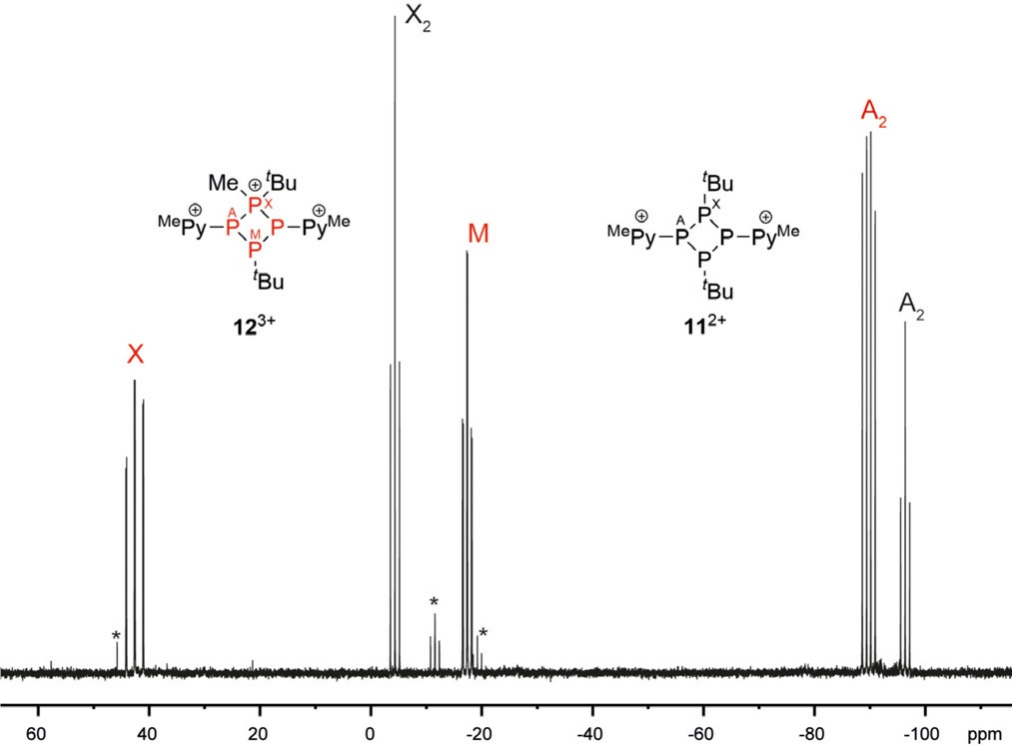
^31^P{^1^H} NMR spectrum of the reaction of **5** with 5 equiv. MeOTf (CD_3_NO_2_; 300 K); small amounts of unidentified compounds are indicated by asterisks.

To access compounds of type **V** (Figure 2) we attempted harsher methylation conditions of **4** and **6** by reacting them in a slurry of a fivefold excess of MeOTf similarly as reported by Burford and co‐workers.12d In case of **4**, only the exclusive formation of the mono‐methylated salt **6**[OTf] is observed with no indication for the formation of other compounds. However, in case of **5** the formation of two distinct cations are observed as indicated by the ^31^P{^1^H} NMR spectrum of the heterogeneous reaction mixture, dissolved in CD_3_NO_2_ (Figure 7).

**Scheme 4:**
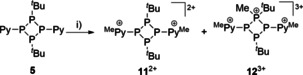
Reaction of **5** with excess MeOTf; i) 5 equiv MeOTf, neat, r.t., 16 h; unbalanced equation.

The presence of an A_2_X_2_ spin system suggests the formation of the *C*_2*v*_ symmetric cation **11** ^2+^ [*δ*(P_A_)=−96.4 ppm, *δ*(P_X_)=−4.3 ppm; ^1^ *J*(P_A_P_X_)=−132 Hz] and the observed A_2_MX spin system [*δ*(P_A_)=−89.8 ppm, *δ*(P_M_)=−17.4 ppm, *δ*(P_X_)=42.6 ppm; ^1^ *J*(P_A_P_X_)=−249 Hz, ^1^ *J*(P_A_P_M_)=−129 Hz, ^2^ *J*(P_M_P_X_)=22 Hz] is significantly different from that of the mono‐methylated cation **8** ^+^ suggesting the formation of tricationic **12** ^3+^ (Scheme 4). We justify our findings by the observed shifts of the A_2_X_2_ spin system which are in the region of tri‐coordinated phosphorus atoms, whereas the resonance at *δ*(P_X_)=42.6 ppm for cation **12** ^3+^ is typical for a tetra‐coordinated phosphorus.^22^ Our assumption was confirmed by X‐ray analysis since we were able to crystalize the salts, however, repeatedly as mixtures. After removal of the excess MeOTf from the reaction mixture, crystals of **11**[OTf]_2_ suitable for X‐ray analysis grew from a MeCN solution by slow vapor diffusion of Et_2_O at −30 °C next to the deposition of copious amounts of amorphous material. If the reaction mixture is dissolved with excess of MeOTf in MeNO_2_ followed by Et_2_O addition by slow vapor diffusion at −30 °C, suitable crystals of **12**[OTf]_3_*2 MeNO_2_ could be harvested.

**Figure 8:**
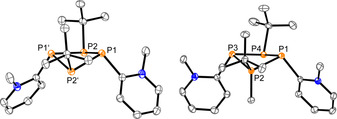
Molecular structures of **11** ^2+^ in **11**[OTf]_2_ (left) and **12** ^3+^ in **12**[OTf]_3_*2 MeNO_2_ (right) (hydrogen atoms, anions and solvate molecules are omitted for clarity, thermal ellipsoids are displayed at 50 % probability); selected bond lengths and angles are given in Table 3.

The separation of **11**[OTf]_2_ from **12**[OTf]_3_ by fractional crystallization was not possible so far, thus, hampering the isolation of analytically pure salts. Figure 8 displays the molecular structures of the cations **11** ^2+^ and **12** ^3+^. The P−P bond lengths in dicationic **11** ^2+^ are comparable to those of tetraphosphetanes **4** and **5** (Table 3). The P‐P‐P angles differ only slightly indicating only a minor influence of the methylation of the pyridyl‐substituents to the structural parameters of the P_4_‐core.

**Figure 9:**
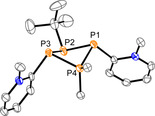
Molecular structure of **13** ^3+^ in **13**[OTf]_3_*MeNO_2_ (hydrogen atoms, solvate molecules and anions are omitted for clarity, thermal ellipsoids are displayed at 50 % probability). Selected bond lengths and angles are given in Table 3.

**Scheme 5:**
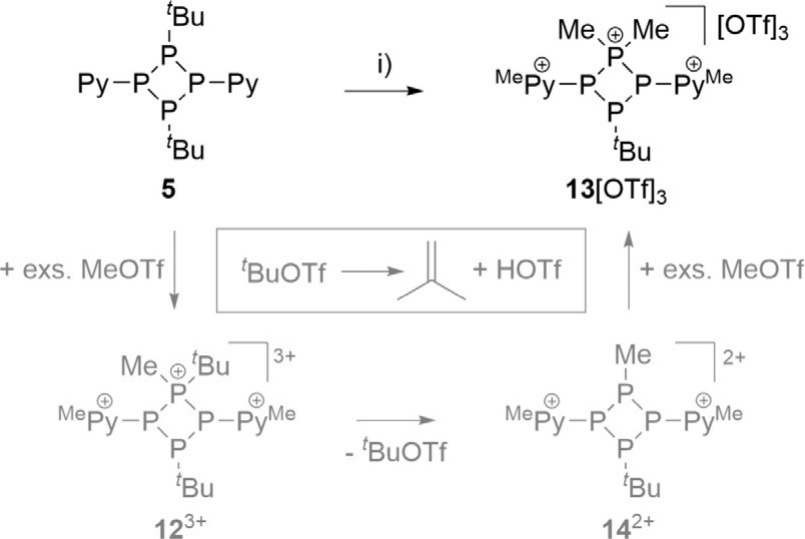
Proposed mechanism of the formation of **13**[OTf]_3_ from the reaction of **5** with MeOTf at elevated temperatures; i) 22 equiv MeOTf, neat, 80 °C, 4 h, *‐* ^*i*^butene, ‐HOTf, 91 %.

Consistent with our findings, the structural parameters of trication **12** ^3+^ are very similar to those of mono‐methylated tetraphosphetanium cations **6** ^+^ and **8^+^** (Table 3). Our attempts to selectively synthesize **12**[OTf]_3_ included the reaction of **5** in a 22‐fold excess of MeOTf at elevated temperature (80 °C; microwave). After 4 h, a red‐colored solution was obtained, and, although the ^31^P{^1^H} NMR spectrum showed a rather complex mixture of compounds, one very dominant A_2_MX spin system [*δ*(P_A_)=−73.5 ppm, *δ*(P_M_)=−12.0 ppm, *δ*(P_X_)=19.9 ppm; ^1^ *J*(P_A_P_X_)=−228 Hz, ^1^ *J*(P_A_P_M_)=−118 Hz, ^2^ *J*(P_M_P_X_)=31 Hz] indicated the formation of a new compound in high yields. After the addition of Et_2_O to the reaction mixture, copious amounts of a colorless precipitate are obtained. Washing with CH_2_Cl_2_ gives the analytically pure product in high yields (91 %) which was identified as **13**[OTf]_3_ after recrystallization from a MeNO_2_ solution and slow vapor diffusion of Et_2_O or CH_2_Cl_2_ at −30 °C (Figure 9). We reason the formation of cation **13** ^3+^ according to Scheme 5 through a series of alkylation and dealkylation reactions in which the first step is the formation of the tri‐methylated trication **12** ^3+^. Due to the high charge of cation **12** ^3+^, the nucleophilic attack of one triflate anion and elimination of *t*BuOTf is supported and leads to the formation of dication **14** ^2+^. At temperatures above −30 °C *t*BuOTf is known to decompose to *iso*butene and HOTf.^23^ The formation of the latter can be traced by multi nuclear NMR spectroscopy of the reaction mixture (*δ*(H)=15.42 ppm (br), *δ*(F)=−76.0 ppm (s)).23b In the last step, dication **14** ^2+^ is regioselectively methylated due to the excess of MeOTf to trication **13** ^3+^. The molecular structure of **13** ^3+^ is shown in Figure 9. The λ^3^P−λ^4^P^+^ bond lengths in **13** ^3+^ (P3−P4 2.203(2) Å, P4−P1 2.204(2) Å) are marginally shorter compared to the λ^3^P−λ^4^P^+^ bond lengths in **12** ^3+^ (P1−P2 2.2034(5) Å and P2−P3 2.2120(5) Å). Moreover, they are considerably shorter than the other P−P bond lengths in **13** ^3+^ (P1−P2 2.247(2) Å, P2−P3 2.238(2) Å) which is caused by the bond polarization in the λ^3^P‐λ^4^P^+^ bond. This leads to a large λ^3^P‐λ^4^P^+^‐λ^3^P bond angle (P3‐P4‐P1 85.44(6)°) compared to the smaller P‐P‐P angles in **13** ^3+^ (P1‐P2‐P3 83.61(6)°, P2‐P3‐P4 81.51(6)°, P4‐P1‐P2 81.29(5)°), which is consistent with our findings for the tetraphosphetan‐1‐ium salts **6**[OTf] and **8**[OTf] (Table 3).

**Scheme 6:**
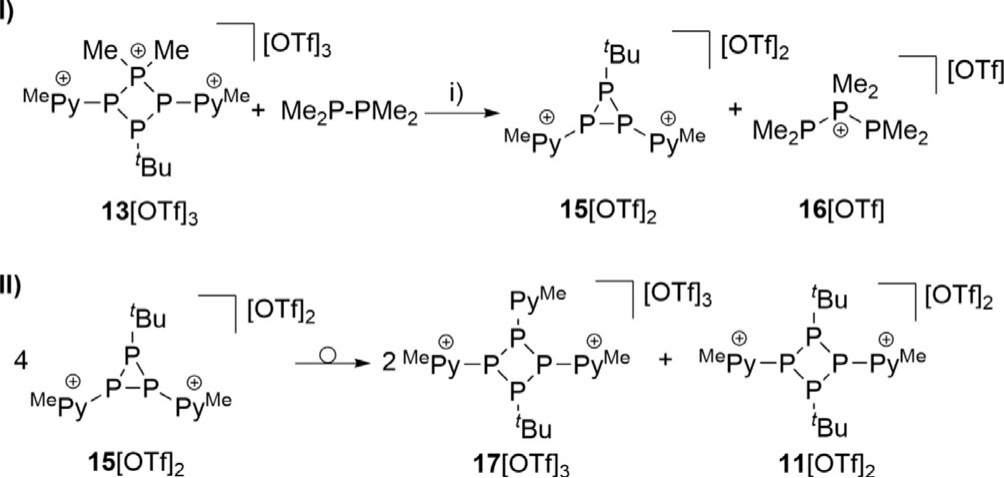
Suggested mechanism of the formation of **17**[OTf]_3_ from the reaction of **13**[OTf]_3_ with Me_2_PPMe_2_ by P−P/P−P bond metathesis (**I**) and a rearrangement reaction of the in situ generated triphosphirane **15** ^2+^ (**II**); i) MeCN, r.t., 4 h, 45 %.

Part of our investigation was, if ring contraction of **13** ^3+^ by phosphenium abstraction gives access to the three‐membered dication **15** ^2+^. Related approaches have been demonstrated by the Burford group which reacted tetraphosphetanium salts of type **IV** (Figure 2) with strong bases such as PMe_3_ and observed the formation of the respective ring contracted triphosphirane and phosphanylphosphonium salts.12a In our reactions we chose the more basic diphosphane Me_2_PPMe_2_ and added an equimolar amount to a solution of **13**[OTf]_3_ in MeCN. A deep red‐colored reaction mixture was observed immediately which showed after a reaction time of 4 h at ambient temperature three main products as judged from the ^31^P{^1^H} NMR spectrum.^24^ Hexamethyltriphosphan‐2‐ium cation **16** ^2+^ can be identified by its characteristic A_2_X spin system [*δ*(P_A_)=−59.2 ppm, *δ*(P_X_)=10.5 ppm; ^1^ *J*(P_A_P_X_)=298 Hz]12a and is the product of the successful phosphenium abstraction from **13** ^3+^.^24^ In addition, the presence of a known A_2_X_2_ spin system identifies the formation of dicationic **11** ^2+^ [*δ*(P_A_)=−96.4 ppm, *δ*(P_X_)=−4.3 ppm; ^1^ *J*(P_A_P_X_)=−132 Hz] and a new A_2_MX spin system [*δ*(P_A_)=−68.3 ppm, *δ*(P_M_)=−51.1 ppm, *δ*(P_X_)=2.7 ppm; ^1^ *J*(P_A_P_X_)=−123 Hz, ^1^ *J*(P_A_P_M_)=−100 Hz, ^2^ *J*(P_M_P_X_)=91 Hz] can be assigned to tricationic **17** ^3+^ (Scheme 6).

**Figure 10:**
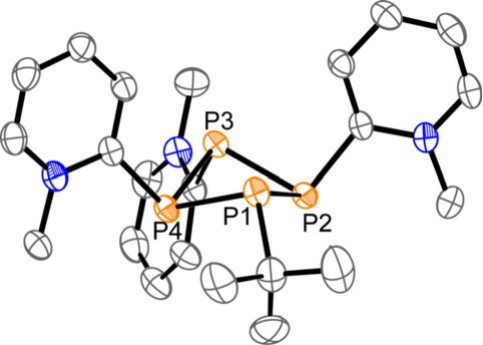
Molecular structure of **17^3+^** in **17**[OTf]_3_ (hydrogen atoms, solvate molecules and anions are omitted for clarity, thermal ellipsoids are displayed at 50 % probability); selected bond lengths and angles are given in Table 3.

At first glance, these findings are different from our assumption, which does not agree with the observation of cation **11** ^2+^ and the new cation **17** ^3+^. In order to shed light on this outcome, we performed the reaction at −30 °C and monitored the progress of the reaction during the warm‐up to room temperature. At −30 °C the ^31^P{^1^H} NMR spectrum shows mainly the resonances for cation **16** ^+^ and an a characteristic A_2_X spin system [*δ*(P_A_)=−138.3 ppm, *δ*(P_X_)=−106.5 ppm; ^1^ *J*(P_A_P_X_)=−199 Hz] which is consistent with a triphosphirane, and thus, we assigned it to dication **15** ^2+^ (Scheme 6, **I**).^24^ During warm‐up, the resonances for **15** ^2+^ completely vanish and the resonances of cation **11** ^2+^ and **17** ^3+^ appear. It is known that cyclophosphanes can undergo interconversion reactions in protic solvents to give thermodynamically more favored ring sizes.^4^ Therefore, we assume that triphosphirane **15** ^2+^ undergoes a ring‐interconversion as a result of a P−P/P−P bond metathesis to form cations **17** ^3+^ and **11** ^2+^ (Scheme 6, **II**).^3^, ^25^ We have been able to separate salt **17**[OTf]_3_ from the reaction mixture in yields of 45 % by slow vapor diffusion of CH_2_Cl_2_ into the reaction mixture at −30 °C. The obtained clear, yellow‐colored crystals were suitable for X‐ray analysis and the molecular structure of **17** ^3+^ is depicted in Figure 10. The P−P bond lengths in **17** ^3+^ are in the expected range of P−P single bonds and comparable to the P−P bonds in **4**, **5** and **11** ^2+^ (Table 3). Compared to **11** ^2+^ (P1‐P2‐P1’ 86.30(2)°, P2‐P1‐P2’ 87.21(2)°) the exchange of one *tert*‐butyl moiety for one methylpyridiniumyl‐substituent leads to smaller P‐P‐P angles in **17** ^3+^ (P1‐P2‐P3 82.92(3)°, P2‐P3‐P4 84.44(3)°, P3‐P4‐P1 83.17(9)°, P4‐P1‐P3 83.88(2)°), as the methylpyridiniumyl group is sterically less demanding.

## Conclusions

In summary, we could show that reacting dipyrazolylphosphanes PhPpyr_2_ and PyPpyr_2_ with *t*BuPH_2_ is a convenient and effective way to synthesize mixed‐substituted tetraphosphetanes **4** and **5** in good yields. Bearing additional donor sites, we eagerly investigated the diverse coordination behavior of pyridyl‐substituted tetraphosphetane **5** towards coinage metal triflate salts. The synthesized coordination complexes were studied by X‐ray analysis and multinuclear NMR spectroscopy at various temperatures. Further reactivity studies of the mixed‐substituted tetraphosphetanes **4** and **5** focused on the reactivity towards electrophilic MeOTf. Next to the expected formation of mono‐methylated tetraphosphetane‐1‐ium triflate salts **6**[OTf] and **8**[OTf], methylation reactions of **5** showed a rich and diverse chemistry due to the additional donor site of the pyridyl‐substituents. While treatment of **5** with excess MeOTf yielded a mixture of di‐ and trimethylated **11^2+^** and **12** ^3+^, additional heating allowed for the synthesis of tricationic **13** ^3+^ through unprecedented alkylation and dealkylation steps. Treating **13** ^3+^ with Me_2_PPMe_2_ leads to **11** ^2+^ and **17** ^3+^ via triphosphirane **15** ^2+^, which is formed in a P−P/P−P bond formation reaction and rearranges to form the thermodynamically favored products **11** ^2+^ and **17** ^3+^. This reaction sequence was intensively studied by multinuclear (2D) NMR spectroscopy. Additionally, all of the methylated tetraphosphetanes were studied by X‐ray analysis, confirming their structural connectivity.

## Experimental Section

**Crystallographic data**: Deposition numbers 1990335, 1990336, 1990337, 1990338, 1990339, 1990340, 1990341, 1990342, 1990343, 1990344, and 1990345 contain the supplementary crystallographic data for this paper. These data are provided free of charge by the joint Cambridge Crystallographic Data Centre and Fachinformationszentrum Karlsruhe Access Structures service.

## Conflict of interest

The authors declare no conflict of interest.

## Supporting information

As a service to our authors and readers, this journal provides supporting information supplied by the authors. Such materials are peer reviewed and may be re‐organized for online delivery, but are not copy‐edited or typeset. Technical support issues arising from supporting information (other than missing files) should be addressed to the authors.

Supplementary
