# Supplementary material for: Coordination Chemistry and Methylation of Mixed‐Substituted Tetraphosphetanes (RP−PtBu)2 (R=Ph, Py)
Source: Chemistry. 2020 Aug 17;26(51):11734–41. doi: 10.1002/chem.202001360 (PMC7540047; doi:10.1002/chem.202001360)
Supplement: Supplementary file 1 — Supplementary [file CHEM-26-11734-s001.pdf]

Chemistry–A European Journal

Supporting Information

Coordination Chemistry and Methylation of Mixed-Substituted Tetraphosphetanes $(RP{-}PtBu)_2$ (R = Ph, Py)

Robin Schoemaker, Philipp Kossatz, Kai Schwedtmann, Felix Hennersdorf, and Jan J. Weigand*[a]

Supporting Information

Coordination Chemistry and Methylation of Mixed-substituted Tetraphosphetanes (RP-P^{t}Bu)$_2$ (R = Ph, Py)

Robin Schoemaker,[a] Philipp Kossatz,[a] Kai Schwedtmann,[a] Felix Hennersdorf[a] and Jan J. Weigand[a*]

a) TU Dresden, Department of Chemistry and Food Chemistry, 01062 Dresden, Germany;
[*] e-mail: jan.weigand@tu-dresden.de

Contents

1. Experimental Section ... 1
1.1. Materials and Methods ... 1
2. Synthesis and spectroscopic data ... 2
2.1. Preparation of bis-(3,5-dimethylpyrazolyl)phenylphosphane 3 ... 2
2.2. Preparation of 2,4-di-tert-butyl-1,3-diphenyltetraphosphetane 4 ... 2
2.3. Preparation of 2,4-di-tert-butyl-1,3-dipyridyltetraphosphetane 5 ... 4
2.4. Preparation of [Cu5$_2$][OTf] ... 5
2.5. Preparation of [Ag5$_2$][OTf] ... 7
2.6. Preparation of [Au5$_2$][OTf] ... 9
2.7. 2,4-Di-*tert*-butyl-2-methyl-1,3-diphenyltetraphosphetan-2-ium triflate 6[OTf] ... 11
2.8. Preparation of 2,4-di-*tert*-butyl-2-methyl-1,3-dipyridyltetraphosphetan-2-ium triflate 8[OTf] ... 12
2.9. Preparation of 1,3-bis(1-methylpyridin-1-ium)-4-(*tert*-butyl)-2,2-dimethyltetraphosphetane-2-ium triflate 13[OTf]$_3$ ... 13
2.10. Preparation of 1,2,3-tris(1-methylpyridin-1-ium)-4-(*tert*-butyl)-tetraphosphetane triflate 17[OTf]$_3$ ... 14
2.11. ^{31}P NMR spectroscopic investigations on the formation of 17^{3+} ... 15
3. X-ray Diffraction Refinements ... 17
4. References ... 22

1. Experimental Section

1.1. Materials and Methods

All manipulations were performed in a Glovebox MB Unilab or using Schlenk techniques under an atmosphere of purified argon or nitrogen. Dry, oxygen-free solvents were distilled either from molecular sieves 3 Å ($MeNO_2$), CaH_2 (CH_2Cl_2, CH_3CN), potassium/benzophenone (Et_2O) or from potassium (*n*-pentane). Anhydrous CD_3CN, CD_3NO_2 and CD_2Cl_2 were purchased from Sigma-Aldrich. With the exception of $MeNO_2$ and CD_3NO_2 all distilled and deuterated solvents were stored over molecular sieves (4 Å: CH_2Cl_2, *n*-pentane, Et_2O, CD_2Cl_2; 3 Å: CD_3CN, CH_3CN). All glassware was oven-dried at 160 °C prior to use. $PyP(Me_2pyr)_2$ **1**,[1] *tert*-butylphosphane[2], tetramethyldiphosphane[3] and (tht)AuCl[4] were prepared as described in the literature. Ag[OTf] and $[Cu(MeCN)_4][OTf]$ were purchased by Sigma Aldrich and used as received. MeOTf and Me_3SiOTf were purchased from Manchester Organics and distilled under inert conditions prior to use. NMR spectra were measured on a Bruker AVANCE III HD Nanobay 400 MHz UltraShield (^{1}H: 400.13 MHz, ^{13}C: 100.61 MHz, ^{31}P: 161.98 MHz, ^{19}F: 376.50 MHz, ^{77}Se: 76.31 Hz), or on a Bruker AVANCE III HDX, 500 MHz Ascend (^{1}H: 500.13 MHz, ^{13}C: 125.75 MHz, ^{31}P: 202.45 MHz, ^{19}F: 470.59 MHz). Reported numbers assigning atoms in the ^{13}C spectra were indirectly deduced from the cross-peaks in 2D correlation experiments (HMBC, HSQC). Chemical shifts are referenced to $\delta(Me_4Si) = 0.00$ ppm (^{1}H, ^{13}C, externally), $\delta(CFCl_3) = 0.00$ ppm (externally) and $\delta(H_3PO_4$, 85%) = 0.00 ppm (externally). Unless stated otherwise, all NMR spectra were measured at 300 K. Chemical shifts (δ) are reported in ppm. Coupling constants (J) are reported in Hz. The designation of the spin systems is performed by convention. The furthest downfield resonance is denoted by the latest letter in the alphabet and the furthest upfield by the earliest letter. Melting points were recorded on an electrothermal melting point apparatus (Büchi Switzerland, Melting point M-560) in sealed capillaries under Nitrogen atmosphere and are uncorrected. Infrared (IR) and Raman spectra were recorded at ambient temperature using a Bruker Vertex 70 instrument equipped with a RAM II module (Nd: YAG laser, 1064 nm). The Raman intensities are reported in percent relative to the most intense peak and are given in parenthesis. An ATR unit (diamond) was used for recording IR spectra. The intensities are reported relative to the most intense peak and are given in parenthesis using the following abbreviations: vw = very weak, w = weak, m = medium, s = strong, vs = very strong. Elemental analyses were performed on a Vario MICRO cube Elemental Analyzer by Elementar Analysatorsysteme GmbH in CHNS modus.

2. Synthesis and spectroscopic data

2.1. Preparation of bis-(3,5-dimethylpyrazolyl)phenylphosphane 3

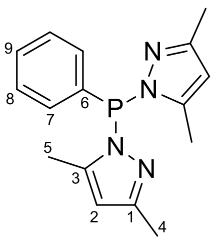

$PhPCl_2$ (6.018 g, 33.62 mmol, 1.00 eq.) was added dropwise to 3,5-dimethyl-1-(trimethylsilyl)pyrazole (13.62 g, 80.94 mmol, 2.41 eq.). The mixture was stirred overnight at ambient temperature. All volatiles were removed *in vacuo* yielding **185** as colourless solid.

Yield: quant.; **mp.:** 70-74 °C; **Raman (80 mW, in cm^{-1}):** 3115(7), 3086(7), 3064(59), 3047(7), 2925(78), 1590(55), 1572(17), 1440(31), 1383(9), 1370(6), 1189(8), 1157(7), 1105(36), 1027(47), 1019(13), 998(100), 743(11), 704(14), 637(14), 616(19), 588(64), 551(13), 483(18), 459(9), 390(13), 375(6), 286(7), 253(8), 241(6), 222(7), 193(17); **IR (ATR, in cm^{-1}):** 2924(w), 1561(w), 1432(w), 1406(w), 1322(vw), 1286(m), 1152(w), 1125(s), 1104(vw), 1018(w), 960(m), 807(m), 762(w), 743(m), 704(m), 688(w), 659(vw), 585(w), 549(m), 503(s), 478(vs), 441(m); **^{1}H NMR (C_6D_6, 300 K, in ppm):** δ = 7.48 (2H, m, C7–H), 7.02 (2H, m, C8–H), 7.00 (1H, m, C9–H), 5.65 (2H, s, C2–H), 2.27 (6H, s, C5–H), 2.12 (6H, s, C4–H); **^{13}C{^{1}H} NMR (C_6D_6, 300 K, in ppm):** δ = 153.1 (2C, d, ^{3}J(CP) = 8.1 Hz, C1). 148.7 (2C, d, ^{2}J(CP) = 12.5 Hz, C3), 136.2 (1C, d, ^{1}J(CP) = 3.3 Hz, C6), 131.6 (2C, d, ^{2}J(CP) = 21.4 Hz, C7), 129.7 (1C, d, ^{4}J(CP) = 1.2 Hz, C9), 128.6 (2C, d, ^{3}J(CP) = 5.4 Hz, C8), 109.1 (2C, s, C2), 14.0 (2C, s, C4), 13.0 (2C, d, ^{3}J(CP) = 11.7 Hz, C5); **^{31}P{^{1}H} NMR (C_6D_6, 300 K, in ppm):** δ = 61.5 (s); **elemental analysis:** calcd for $C_{16}H_{19}N_4P$: C: 64.4, H: 6.4, N: 18.8, found: C:64.3, H: 6.5, N: 19.1.

2.2. Preparation of 2,4-di-tert-butyl-1,3-diphenyltetraphosphetane 4

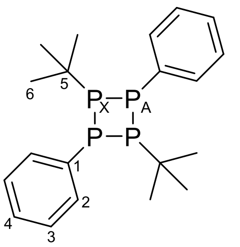

To a solution of *t*$BuPH_2$ (700 mg, 7.77 mmol, 1.00 ml) in 10 ml of MeCN phenyldipyrazolylphosphan **3** (2.213 g, 7.77 mmol) in 10 ml of MeCN is added at -45 °C. The reaction mixture is stored at -30 °C for 16 h to give a colorless suspension. All volatiles of this suspension are evaporated *in vacuo* to give a colorless residue. After sublimation of 3,5-dimethylpyrazole from the residue (80 °C; 6 x 10^{-3} mbar) compound **4** is obtained quantitatively in a purity of >95% (see figure S2.1; bottom). Analytically pure **4** (see figure S2.1; top) is obtained by washing the crude product with cold MeCN (-30 °C; 3 x 2 ml) and subsequent evaporation of all volatiles *in vacuo*.

Yield: 1.113 g (69%); **m.p.:** 96 °C; **Raman (100 mW, 298 K, in cm^{-1}):** ν = 3058 (32), 3046 (36), 2962 (25), 2944 (24), 2930 (25), 2915 (31), 2889 (39), 2854 (25), 1582 (61), 1455 (19), 1443 (14), 1283 (5), 1272 (8), 1199 (8), 1180 (18), 1157 (14), 1085 (20), 1064 (9), 1026 (33), 1014 (6), 999 (100), 936 (14), 806 (18), 691 (8), 618 (11), 575 (18), 486 (23), 471 (44), 241 (17), 207 (32), 178 (45), 167 (38), 130 (74); **IR (ATR, 298 K, in cm^{-1}):** ν = 3059 (w), 2943 (m), 2929 (m), 2887 (w), 2852 (m), 1580 (w), 1520 (w), 1469 (m), 1455 (m), 1433 (m), 1400 (w), 1387 (w), 1359 (s), 1323 (w), 1300 (w), 1282 (m), 1168 (s), 1155 (s), 1099 (w), 1081 (w), 1064 (m), 1024 (s), 998 (m), 934 (m), 911 (w), 805 (m), 742 (vs), 691 (vs), 610 (m), 573 (m), 541 (m), 508 (s), 480 (m), 440 (m), 428 (m), 406 (m); **^{1}H NMR (CD_2Cl_2, 300 K, in ppm):** δ =1.28-1.31 (18H, m, C6–H), 7.28-7.31 (2H, m, C4–H), 7.33-7.37 (4H, m, C3–H), 7.72-7.76 (4H, m, C2–H); **^{13}C{^{1}H} NMR (CD_2Cl_2, 300 K, in ppm):** δ = 28.4-28.6 (6C, m, C6), 31.5-31.9 (2C, m, C5), 128.7 (2C, m, C4), 128.9 (4C, m, C3), 133.8-134.1 (4C, m, C2), 140.3-141.2 (2C, m, C1); **^{31}P NMR (CD_2Cl_2, 300 K, in ppm):** A_2X_2 spin system: δ(P_A) = -88.4 (2P), δ(P_X) = -15.5 (2P); $^{1}J(P_AP_X)$ = -130 Hz; **elemental analysis**: calcd. for $C_{20}H_{28}P_4$: C: 61.23, H: 7.19; found: C: 60.80, H: 7.23.

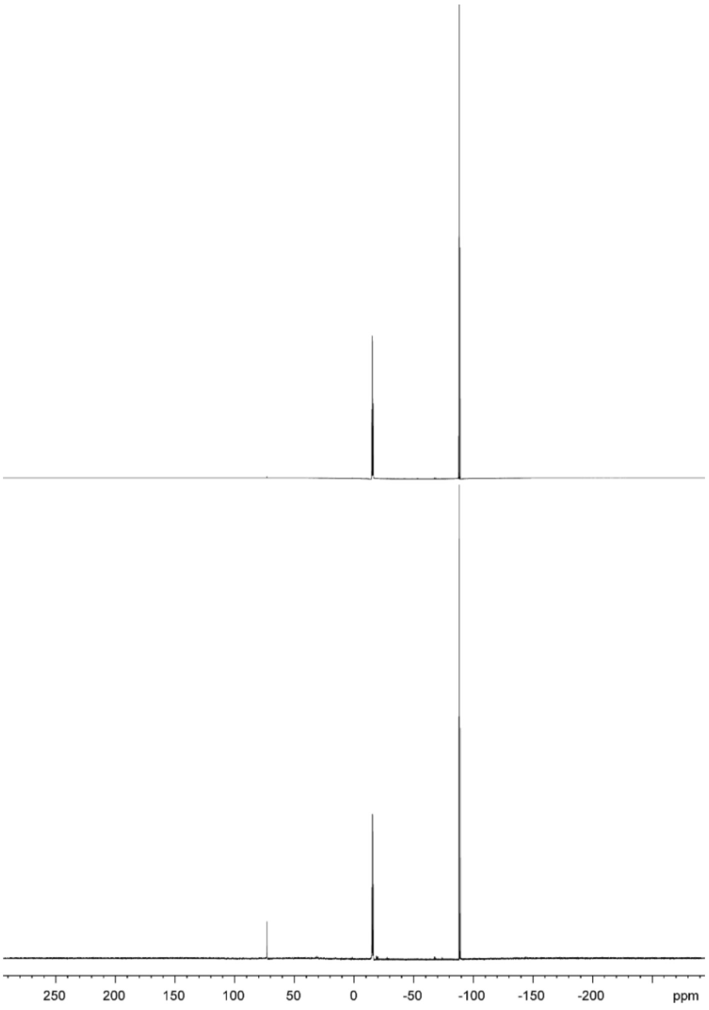

Figure S2.1. ^{31}P NMR spectra of crude $tBu_2Ph_2P_4$ (bottom) and analytically pure $tBu_2Ph_2P_4$ (top).

2.3. Preparation of 2,4-di-tert-butyl-1,3-dipyridyltetraphosphetane 5

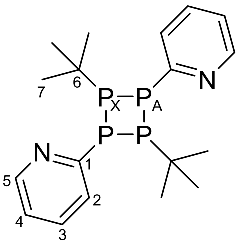

To a solution of *t*BuPH_2 (700 mg, 7.77 mmol, 1.00 ml) in 10 ml of MeCN Pyridyldipyrazolylphosphan **1** (2.326 g, 7.77 mmol) in 20 ml of MeCN is added at -45 °C. The reaction mixture is stored at -30 °C for 16 h to give a colorless suspension. All volatiles of this suspension are evaporated *in vacuo* to give a colorless residue. After sublimation of 3,5-dimethylpyrazole from the residue (80 °C; 6 x 10^{-3} mbar) compound **5** is obtained quantitatively in a purity of >95% (see figure S2.2; bottom). Analytically pure **5** (see figure S2.2; top) is obtained by washing the crude product with cold MeCN (-30 °C; 3 x 2 ml) and subsequent evaporation of all volatiles *in vacuo*.

Yield: 860 mg (53%); **m.p.:** 173 °C; **Raman (100 mW, 298 K, in cm^{-1}):** v = 3134 (5), 3119 (9), 3060 (23), 3039 (63), 2950 (66), 2927 (53), 2918 (54), 2890 (90), 2855 (53), 2769 (8), 2704 (10), 1571 (74), 1559 (34), 1458 (29), 1440 (21), 1419 (15), 1389 (6), 1276 (10), 1201 (12), 1174 (15), 1158 (16), 1127 (25), 1084 (7), 1048 (58), 987 (100), 936 (17), 807 (31), 714 (15), 619 (12), 579 (30), 500 (28), 479 (24), 468 (41), 424 (10), 397 (14), 384 (11), 247 (16), 204 (42), 174 (28), 164 (37), 124 (49); **IR (ATR, 298 K, in cm^{-1}):** v = 3036 (w), 2943 (m), 2927 (m), 2888 (m), 2853 (m), 2709 (w), 1569 (vs), 1556 (s), 1470 (m), 1447 (vs), 1416 (vs), 1388 (m), 1360 (vs), 1268 (m), 1227 (m), 1202 (m), 1167 (s), 1149 (s), 1082 (m), 1046 (m), 1008 (m), 986 (m), 934 (w), 887 (w), 804 (m), 768 (s), 757 (vs), 740 (s), 712 (m), 639 (w), 618 (s), 574 (m), 554 (w), 517 (m), 500 (s), 467 (m), 407 (m); **^{1}H NMR (CD_2Cl_2, 300 K, in ppm):** δ =1.36-1.40 (18H, m, C7–H), 7.06-7.10 (2H, m, C4–H), 7.53-7.58 (2H, m, C3–H), 7.72-7.76 (2H, m, C2–H), 8.53-8.57 (2H, m, C5–H); **^{13}C{^{1}H} NMR (CD_2Cl_2, 300 K, in ppm):** δ = 28.4-28.7 (6C, m, C7), 30.9-31.8 (2C, m, C6), 121.9 (2C, s, C4), 127.5-127.8 (2C, m, C2), 135.8 (2C, m, C3), 150.3-150.4 (2C, m, C5), 167.4-167.8 (2C, m, C1); **^{31}P NMR (CD_2Cl_2, 300 K, in ppm):** A_2X_2 spin system: δ(P_A) = -81.9 (2P), δ(P_X) = -18.8 (2P); $^{1}J(P_AP_X)$ = -131 Hz; **elemental analysis**: calcd. for $C_{18}H_{26}N_2P_4$: C: 54.83, H: 6.65, N: 7.10; found: C: 54.60, H: 6.59, N: 7.21.

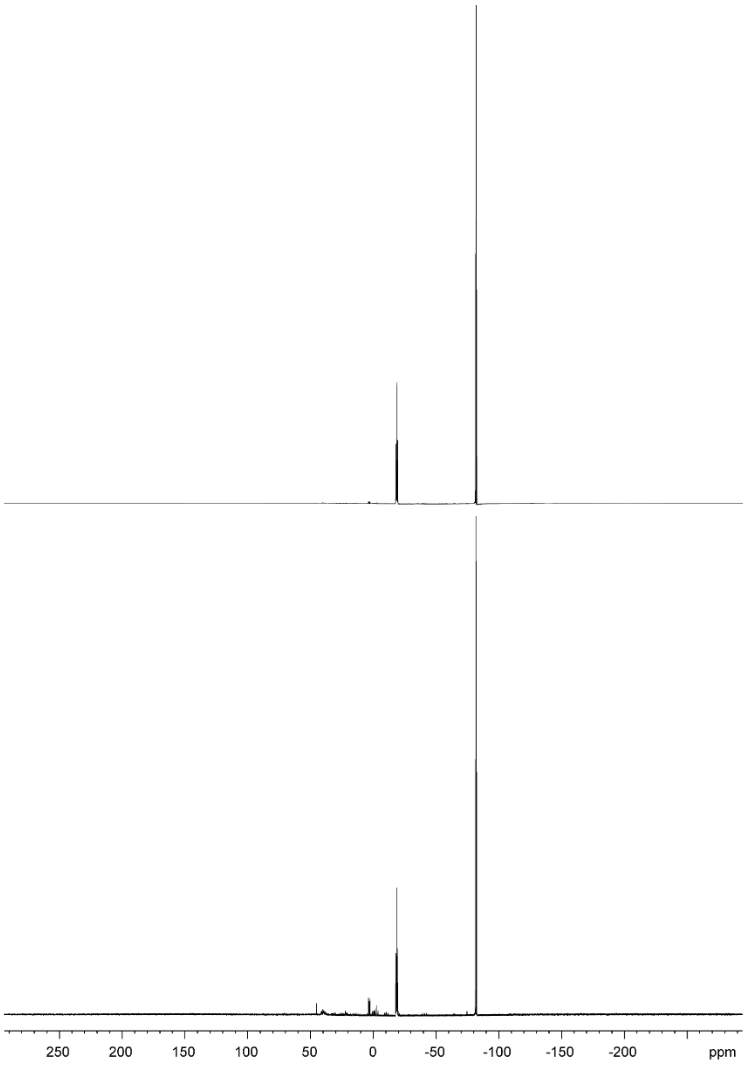

Figure S2.2. ^{31}P NMR spectra of crude *t*$Bu_2Py_2P_4$ (bottom) and analytically pure *t*$Bu_2Py_2P_4$ (top)

2.4. Preparation of [Cu**5**$_2$][OTf]

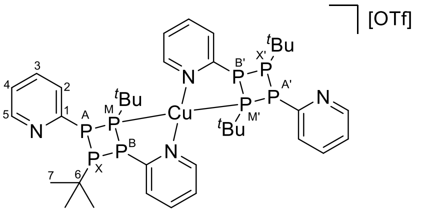

To a solution of Tetraphosphetane **5** (50 mg, 0.13 mmol) in 1 ml of CH_2Cl_2 [Cu(MeCN)$_4$][OTf] (24 mg, 0.065 mmol) dissolved in 0.5 ml CH_2Cl_2 is added. The reaction mixture is stirred for 1 h at room temperature. Vapor diffusion of *n*-pentane into this solution at -30 °C yields yellow crystals of [Cu**5**$_2$][OTf]. These are isolated by decantation, washing with *n*-pentane (3 x 1 ml) and evaporation of all volatiles *in vacuo*.

Yield: 56 mg (89%); **m.p.:** 273 (dec.) °C; **Raman (100 mW, 298 K, in cm^{-1}):** ν = 3120 (15), 3066 (43), 3048 (49), 2992 (23), 2957 (71), 2934 (78), 2919 (69), 2893 (100), 2857 (59), 2775 (11), 2711 (16), 1579 (30), 1572 (39), 1558 (35), 1456 (26), 1445 (25), 1421 (14), 1393 (8), 1275 (9), 1224 (9), 1199 (13), 1158 (15), 1120 (21), 1090 (13), 1052 (25), 1043 (23), 1030 (20), 1006 (51), 986 (33), 935 (9), 805 (21), 755 (9), 714 (13), 702 (9), 588 (19), 513 (43), 498 (16),

487 (10), 471 (26), 447 (14), 398 (11), 364 (8); **IR (ATR, 298 K, in cm^{-1}):** ν = 3066 (w), 3035 (w), 2984 (w), 2950 (w), 2931 (w), 2892 (w), 2857 (w), 1577 (m), 1572 (m), 1557 (w), 1455 (s), 1449 (m), 1419 (m), 1390 (w), 1362 (m), 1260 (vs), 1223 (m), 1148 (vs), 1090 (w), 1051 (w), 1042 (w), 1028 (vs), 1004 (m), 985 (m), 912 (w), 883 (w), 804 (w), 784 (m), 768 (m), 758 (vs), 738 (s), 715 (w), 700 (m), 636 (vs), 618 (m); **^{1}H NMR (CD_2Cl_2, 300 K, in ppm):** δ =1.57 (36H, d, $^{1}J_{HP}$ = 15.1 Hz, C7–H), 6.68-6.72 (4H, m, C4–H), 7.38-7.45 (4H, m, C3–H), 7.52-7.56 (4H, m, C2–H), 7.98-8.02 (4H, m, C5–H); **^{13}C{^{1}H} NMR (CD_2Cl_2, 300 K, in ppm):** δ = 27.3 (12C, dt, $^{1}J_{CP}$ = 11.1 Hz, $^{2}J_{CP}$ = 5.7 Hz, C7), 32.2-32.7 (4C, m, C6), 121.1 (1C, q, $^{1}J_{CF}$ = 321.1 Hz, CF_3), 122.6 (4C, br, C4), 127.0-127.6 (4C, m, C2), 136.3 (4C, br, C3), 150.7-151.2 (4C, m, C5), 162.0-162.6 (4C, m, C1); **^{19}F NMR (CD_2Cl_2, 300 K, in ppm):** δ = –78.9 (3F, s); **^{31}P NMR (CD_2Cl_2, 190 K, in ppm):** AA'BB'MM'XX' spin system δ(P) = δ(P_A) = -81.4 ppm, δ(P_B) = -68.9 ppm, δ(P_M) = -45.1 ppm, δ(P_X) = -15.4 ppm; **elemental analysis**: calcd. for $C_{37}H_{52}CuF_3N_4O_3P_8S$ x 0.75 CH_2Cl_2: C: 42.58, H: 5.06, N: 5.26, S: 3.01; found: C: 42.66, H: 4.93, N: 5.30, S: 3.17.

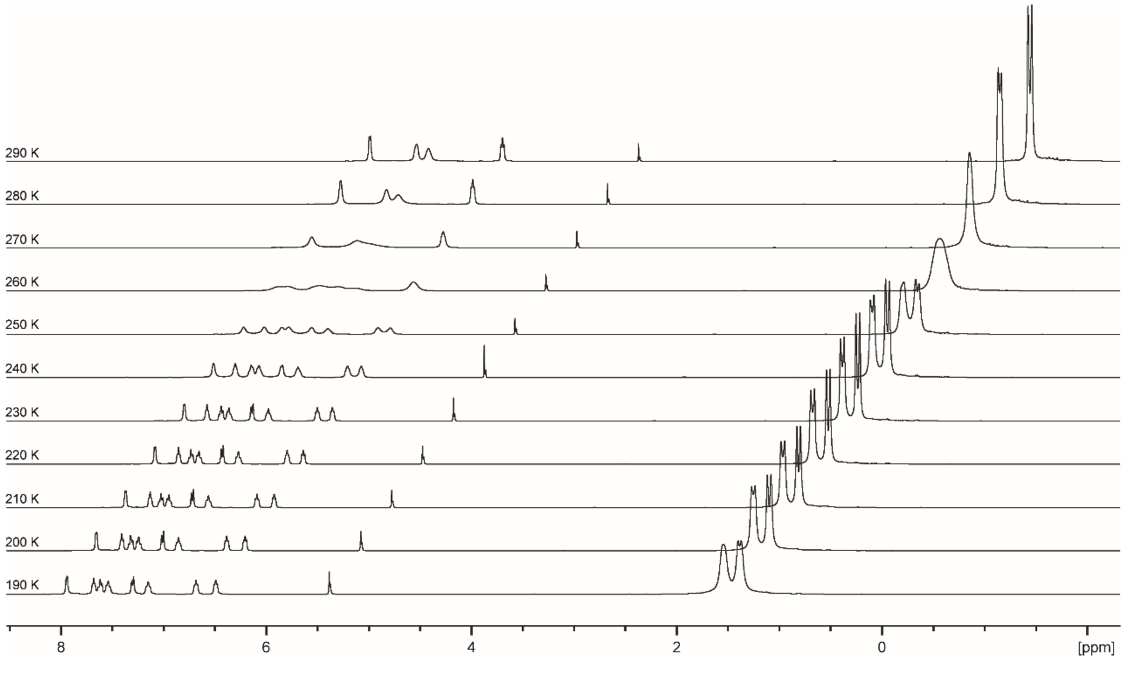

Figure S2.3. ^{1}H NMR spectra of [Cu**5**$_2$][OTf] in CD_2Cl_2 from 190-290 K.

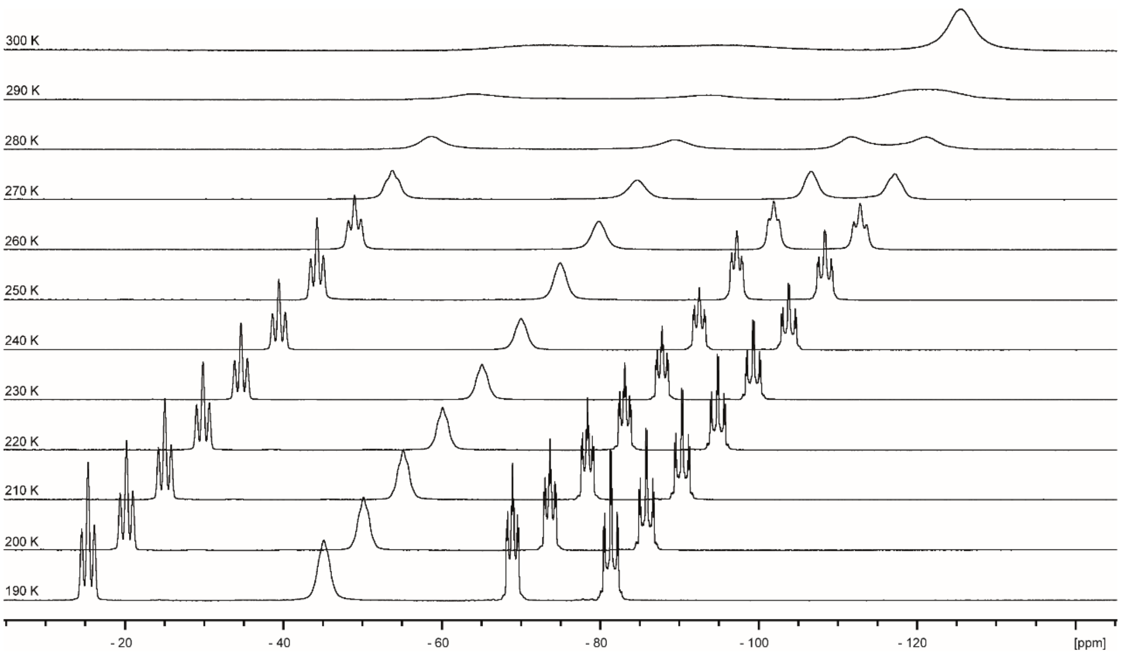

Figure S2.4. ^{31}P NMR spectra of [Cu**5**$_2$][OTf] in CD_2Cl_2 from 190-300 K.

2.5. Preparation of [Ag5$_2$][OTf]

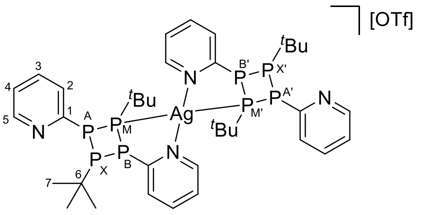

To a solution of tetraphosphetane **5** (79 mg, 0.2 mmol) in 1 ml of CH_2Cl_2 Ag[OTf] (26 mg, 0.1 mmol) dissolved in 0.5 ml CH_2Cl_2 is added. The reaction mixture is stirred for 1 h at room temperature. Vapor diffusion of *n*-pentane into this solution at -30 °C yields colorless crystals of [Ag**5**$_2$][OTf]. These are isolated by decantation, washing with *n*-pentane (3 x 1 ml) and evaporation of all volatiles *in vacuo*. Crystals suitable for X-ray analysis are obtained by recrystallization from MeCN/Et_2O at -30 °C.

Yield: 79 mg (76%); **m.p.:** 247 °C (dec.); **Raman (100 mW, 298 K, in cm^{-1}):** ν = 3119 (13), 3061 (43), 3042 (34), 2955 (74), 2933 (75), 2919 (62), 2894 (100), 2857 (62), 2775 (13), 2711 (21), 1572 (75), 1559 (47), 1458 (38), 1443 (34), 1422 (19), 1392 (13), 1364 (9), 1274 (13), 1225 (15), 1201 (19), 1171 (26), 1129 (26), 1114 (28), 1087 (21), 1046 (72), 1032 (40), 1014 (13), 998 (72), 985 (62), 936 (19), 805 (42), 768 (9), 755 (19), 740 (9), 712 (23), 629 (13), 618 (13), 594 (30), 576 (28), 515 (55), 497 (40), 489 (26), 471 (57), 446 (28); **IR (ATR, 298 K, in cm^{-1}):** ν = 3066 (w), 3037 (vw), 2950 (w), 2930 (w), 2891 (w), 2856 (w), 1572 (m), 1558 (w), 1458 (m), 1448 (m), 1418 (m), 1391 (w), 1362 (w), 1262 (vs), 1223 (m), 1150 (s), 1088 (w), 1045 (w), 1029 (vs), 997 (m), 985 (w), 883 (vw), 804 (w), 783 (m), 759 (vs), 739 (m), 713 (w), 701 (w), 637 (vs), 628 (s); **^{1}H NMR (CD_2Cl_2, 300 K, in ppm):** δ =1.43-1.48 (36H, m, C7–H), 6.82-6.87 (4H, m, C4–H), 7.47-7.54 (4H, m, C3–H), 7.61-7.67 (4H, m, C2–H), 7.98-8.02 (4H,

m, C5–H); **$^{13}C\{^1H\}$ NMR (CD_2Cl_2, 300 K, in ppm):** δ = 27.4-27.6 (12C, m, C7), 32.1-32.4 (4C, m, C6), 121.1 (1C, q, $^1J_{CF}$ = 321.2 Hz), 123.0 (4C, s, C4), 128.0-128.9 (4C, m, C2), 136.4 (4C, *pseudo*-t. J_{CP} = 4.4 Hz, C3), 151.3 (4C, br, C5), 161.2-161.7 (4C, m, C1); **^{19}F NMR (CD_2Cl_2, 300 K, in ppm):** δ = –78.9 (3F, s); **^{31}P NMR (CD_2Cl_2, 190 K, in ppm):** AA'BB'MM'XX' spin system: δ(P_A) = -87.6 ppm, δ(P_B) = -82.4 ppm, δ(P_M) = -32.5 ppm, δ(P_X) = -12.4 ppm; **elemental analysis**: calcd. for $C_{37}H_{52}AgF_3N_4O_3P_8S$ x 1.0 CH_2Cl_2: C: 40.37, H: 4.81, N: 4.96, S: 2.84; found: C: 40.32, H: 4.53, N: 5.10, S: 3.20.

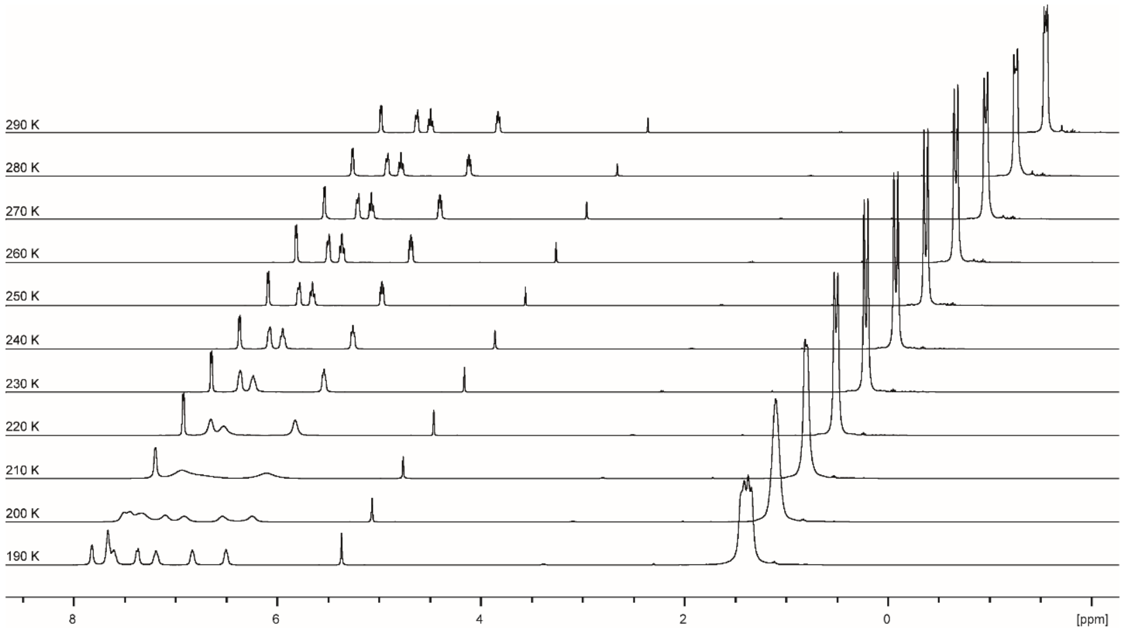

Figure S2.5. ^{1}H NMR spectra of [Ag**5**$_2$][OTf] in CD_2Cl_2 from 190-290 K

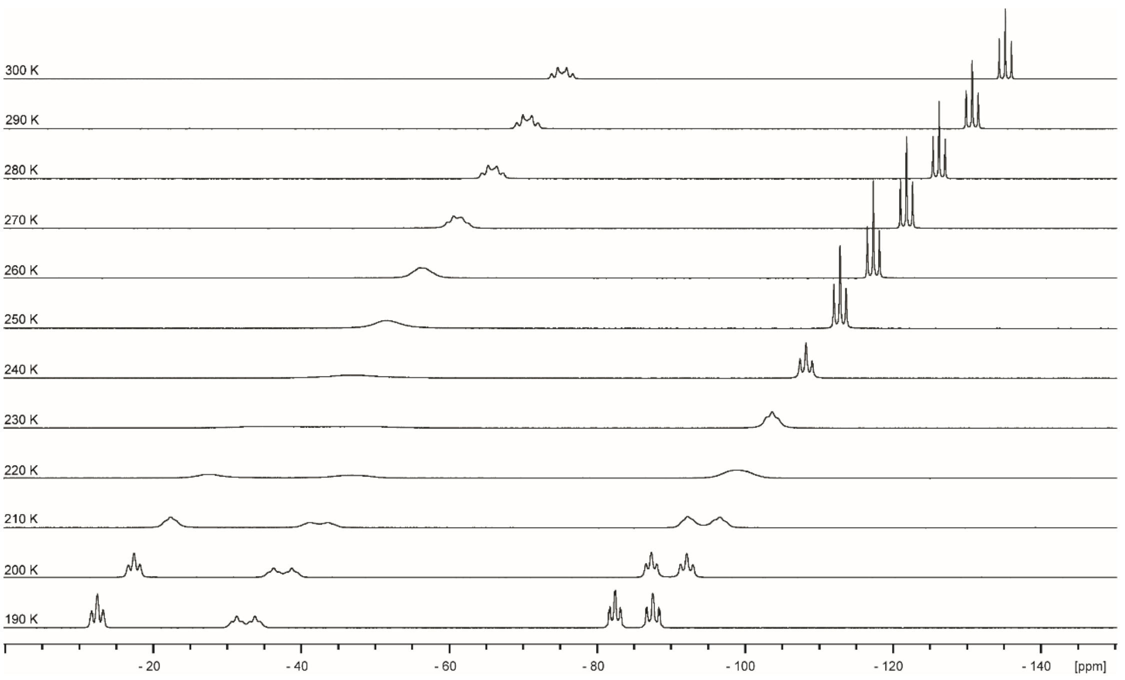

Figure S2.6. ^{31}P NMR spectra of [Ag**5**$_2$][OTf] in CD_2Cl_2 from 190-300 K.

2.6. Preparation of [Au5$_2$][OTf]

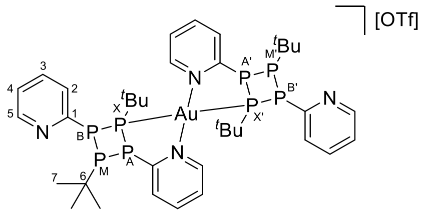

To a solution of tetraphosphetane **5** (87 mg, 0.22 mmol) in 1 ml of CH_2Cl_2 (tht)AuCl (35 mg, 0.11 mmol) dissolved in 0.5 ml CH_2Cl_2 is added, followed by the addition of Me_3SiOTf (20 µl, 0.11 mmol). The reaction mixture is stirred for 1 h at room temperature. Vapor diffusion of *n*-pentane into this solution at -30 °C yields colorless crystals of [Au**5**$_2$][OTf]. These are isolated by decantation, washing with *n*-pentane (3 x 1 ml) and evaporation of all volatiles *in vacuo*.

Yield: 93 mg (74%); **m.p.:** 217 (dec.) °C; **Raman (100 mW, 298 K, in cm^{-1}):** v = 3120 (19), 3062 (46), 3040 (41), 2957 (80), 2934 (74), 2919 (70), 2894 (100), 2857 (64), 2775 (26), 2711 (31), 1570 (80), 1559 (69), 1458 (58), 1443 (52), 1423 (36), 1275 (34), 1200 (36), 1172 (43), 1113 (49), 1086 (39), 1044 (98), 1031 (57), 991 (82), 986 (86), 936 (32), 805 (52), 755 (34), 713 (39), 702 (33), 624 (30), 598 (43), 576 (45), 537 (63), 496 (47), 486 (37), 473 (61), 445 (42), 436 (39), 391 (43), 364 (34), 347 (35), 313 (42); **IR (ATR, 298 K, in cm^{-1}):** v = 3063 (w), 3037 (w), 2953 (w), 2930 (w), 2891 (w), 2856 (w), 1569 (m), 1458 (m), 1445 (m), 1419 (m), 1391 (w), 1362 (m), 1262 (vs), 1223 (m), 1149 (vs), 1086 (w), 1042 (w), 1029 (vs), 988 (m), 937 (w), 883 (w), 804 (w), 782 (m), 759 (vs), 738 (s), 714 (w), 700 (w), 637 (vs), 623 (s); **^{1}H NMR (CD_2Cl_2, 300 K, in ppm):** δ =1.35-1.40 (36H, m, C7–H), 6.94-6.98 (4H, m, C4–H), 7.45-7.50 (4H, m, C3–H), 7.55-7.59 (4H, m, C2–H), 8.23-8.25 (4H, m, C5–H); **^{13}C{^{1}H} NMR**

(CD_2Cl_2, 300 K, in ppm): δ = 27.9-28.3 (12C, m, C7), 33.6-35.8 (4C, m, C6), 121.7 (1C, q, $^1J_{CF}$ = 320.1 Hz), 123.7 (4C, s, C4), 128.3-129.1 (4C, m, C2), 136.8 (4C, *pseudo*-t. J_{CP} = 3.2 Hz, C3), 151.5 (4C, br, C5), 161.3-161.7 (4C, m, C1); **^{19}F NMR (CD_2Cl_2, 300 K, in ppm):** δ = –78.8 (3F, s); **^{31}P NMR (CD_2Cl_2, 190 K, in ppm):** AA'BB'MM'XX' spin system δ(P) = δ(P_A) = -77.3, δ(P_B) = -73.0, δ(P_M) = -17.5, δ(P_X) = 19.2; ^4J($P_AP_{A'}$) = -0.01 Hz, ^2J(P_AP_B) = 3.95 Hz, ^4J($P_AP_{B'}$) = 20.96 Hz, ^1J(P_AP_M) = -135.52 Hz, ^5J($P_AP_{M'}$) = -2.35 Hz, ^1J(P_AP_X) = 165.95 Hz, ^4J($P_AP_{X'}$) = -0.92 Hz, ^4J($P_{A'}P_B$) = 26.27 Hz, ^2J($P_{A'}P_{B'}$) = -4.31 Hz, ^5J($P_{A'}P_M$) = 2.64 Hz, ^1J($P_{A'}P_{M'}$) = -134.40 Hz, ^4J($P_{A'}P_X$) = 0.87 Hz, ^1J($P_{A'}P_{X'}$) = 167.77 Hz, ^4J($P_BP_{B'}$) = -1.19 Hz, ^1J(P_BP_M) = -121.33 Hz, ^5J($P_BP_{M'}$) = 0.18 Hz, ^1J(P_BP_X) = 153.18 Hz, ^3J($P_BP_{X'}$) = -5.67 Hz, ^5J($P_{B'}P_M$) = -0.03 Hz, ^1J($P_{B'}P_{M'}$) = -121.46 Hz, ^3J($P_{B'}P_X$) = 5.18 Hz, ^1J($P_{B'}P_{X'}$) = 142.87 Hz, ^6J($P_MP_{M'}$) = 4.55 Hz, ^2J(P_AP_X) = 7.50 Hz, ^4J($P_MP_{X'}$) = -20.44 Hz, ^4J($P_{M'}P_X$) = -25.96 Hz, ^2J($P_{M'}P_{X'}$) = -0.55 Hz, ^2J($P_XP_{X'}$) = 250.91 Hz; **elemental analysis**: calcd. for $C_{37}H_{52}AuF_3N_4O_3P_8S$ x 1.0 CH_2Cl_2: C: 37.42, H: 4.46, N: 4.59, S: 2.63; found: C: 37.51, H: 4.50, N: 4.50, S: 2.74.

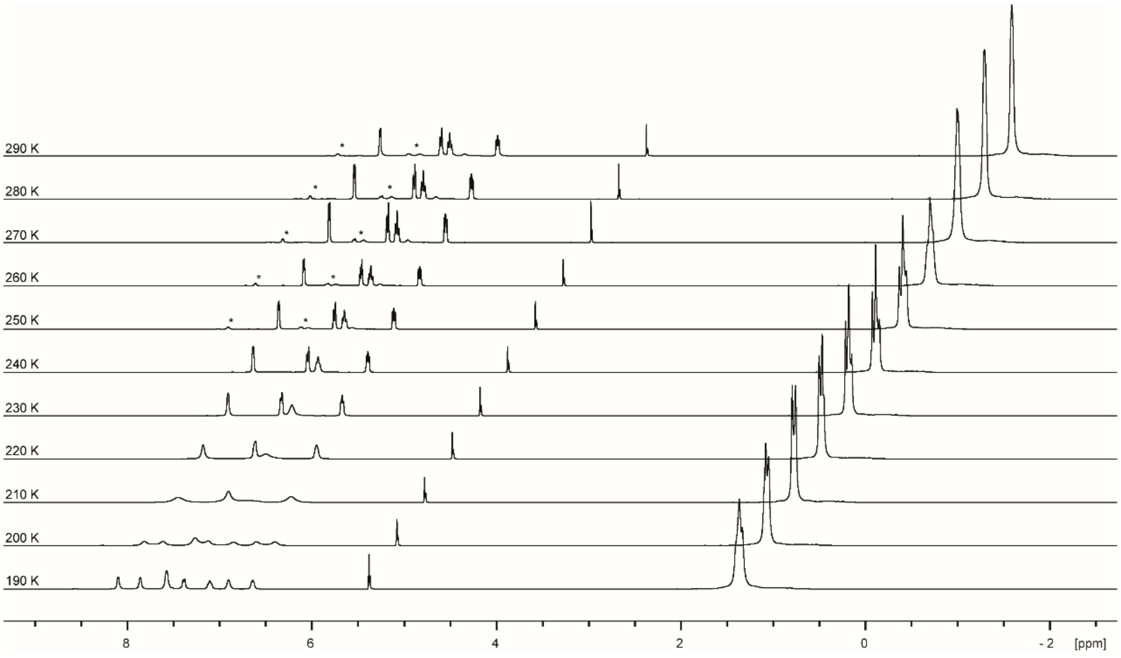

Figure S2.7. ^{1}H NMR spectra of [Au**5**$_2$][OTf] in CD_2Cl_2 from 190-290 K; asterisks indicate small amounts of impurities.

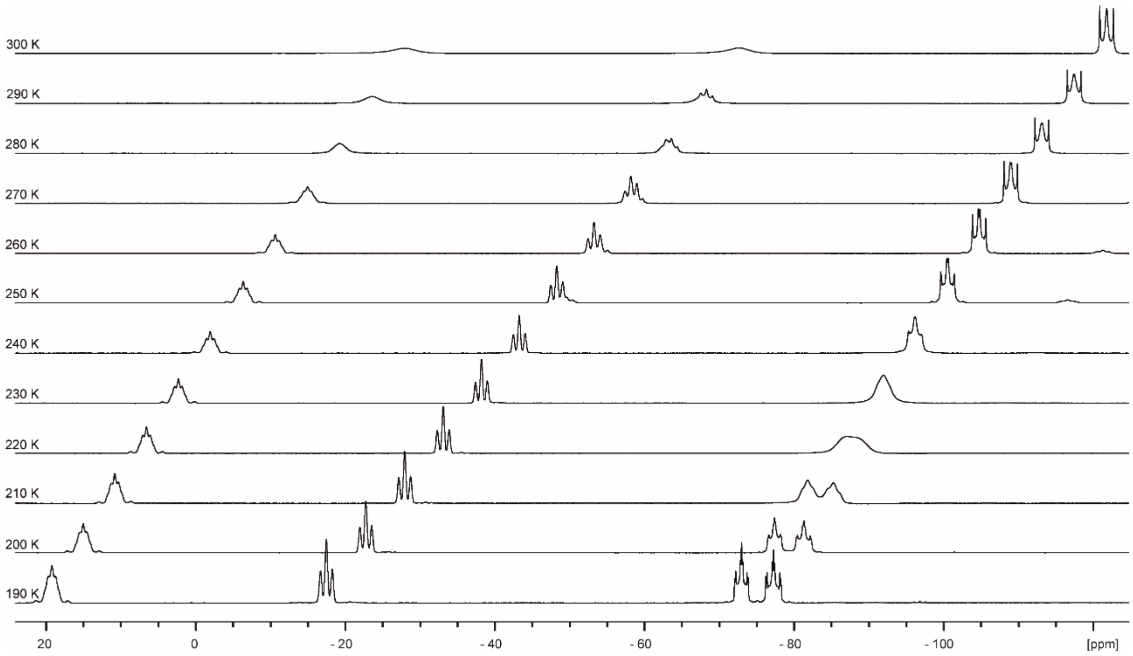

Figure S2.8. ^{31}P NMR spectra of [Au**5**$_2$][OTf] in CD_2Cl_2 from 190-300 K.

2.7. 2,4-Di-*tert*-butyl-2-methyl-1,3-diphenyltetraphosphetan-2-ium triflate 6[OTf]

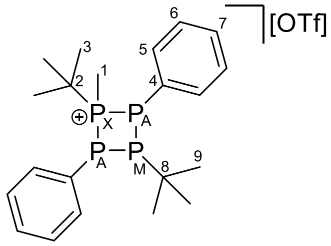

To tetraphosphetane **4** (100 mg, 0.25 mmol) in 2 ml Et_2O MeOTf (41 mg, 0.25 mmol) is added yielding a colorless suspension. After stirring for 16 h at r.t. the suspension is filtered and the residue is washed with Et_2O (2 x 2 ml). Subsequent recrystallization of the residue from MeCN/Et_2O yields crystals of **6**[OTf] which are isolated.

Yield: 124 mg (87%); **m.p.:** 185 °C; **Raman (100 mW, 298 K, in cm^{-1}):** ν = 3057 (46), 2958 (53), 2911 (60), 2896 (52), 1581 (79), 1461 (20), 1440 (19), 1394 (11), 1275 (11), 1224 (14), 1165 (22), 1083 (31), 1025 (60), 1000 (100), 944 (14), 804 (26), 753 (22), 694 (14), 610 (16), 574 (27), 475 (43), 454 (19), 426 (17), 398 (17), 375 (19), 346 (21), 310 (23), 293 (20), 233 (35), 208 (27), 177 (40); **IR (ATR, 298 K, in cm^{-1}):** ν = 3059 (w), 2943 (m), 2929 (m), 2887 (w), 2852 (m), 1580 (w), 1520 (w), 1469 (m), 1455 (m), 1433 (m), 1400 (w), 1387 (w), 1359 (s), 1323 (w), 1300 (w), 1282 (m), 1168 (s), 1155 (s), 1099 (w), 1081 (w), 1064 (m), 1024 (s), 998 (m), 934 (m), 911 (w), 805 (m), 742 (vs), 691 (vs), 610 (m), 573 (m), 541 (m), 508 (s), 480 (m), 440 (m), 428 (m), 406 (m); **^{1}H NMR (CD_2Cl_2, 300 K, in ppm):** δ =1.26 (3H, d, $^{2}J_{HP}$ = 12.11 Hz, C1–H), 1.40 (9H, s, C3–H), 1.42-1.44 (9H, m, C9–H), 7.60-7.64 (6H, m, C6/C7–H), 7.80-7.85 (4H, m, C5–H); **^{13}C{^{1}H} NMR (CD_2Cl_2, 300 K, in ppm):** δ = 3.2-3.4

(1C, m, C1), 24.5 (3C, m, C3), 28.6 (3C, dt, $^2J_{CP}$ = 15.4 Hz, $^3J_{CP}$ = 5.6 Hz, C9), 32.6-33.2 (1C, m, C8), 35.8-36.1 (1C, m, C2), 121.5 (1C, q, $^1J_{CF3}$ = 320.3 Hz, CF_3), 123.7-124.2 (2C, m, C4), 131.0-131.1 (4C, m, C6), 132.6-132.7 (2C, m, C7), 135.5-135.8 (4C, m, C5); **^{19}F NMR (CD_2Cl_2, 300 K, in ppm):** δ = –79.5 (3F, s); **^{31}P NMR (CD_2Cl_2, 300 K, in ppm):** A_2MX spin system: δ(P_A) = -81.2 (2P), δ(P_M) = -39.8 (1P), δ(P_X) = 22.0 (1P); $^1J(P_AP_X)$ = -248 Hz, $^1J(P_AP_M)$ = -127 Hz, $^2J(P_MP_X)$ = 23 Hz; **elemental analysis**: calcd. for $C_{22}H_{31}F_3O_3P_4S$: C: 47.49, H: 5.62, S: 5.76; found: C: 47.28, H: 5.39, S: 6.16.

2.8. Preparation of 2,4-di-*tert*-butyl-2-methyl-1,3-dipyridyltetraphosphetan-2-ium triflate 8[OTf]

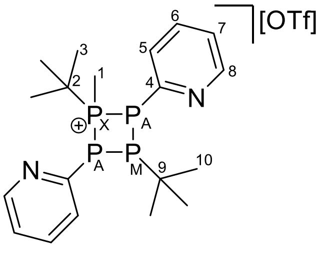

To tetraphosphetane **5** (360 mg, 0.91 mmol) in 3 ml of Et_2O MeOTf (150 mg, 0.91 mmol) is added yielding a colorless suspension. After stirring for 16 h at r.t. the suspension is filtered and the residue is washed with Et_2O (2 x 2 ml). Subsequent recrystallization of the residue from MeCN/Et_2O yields crystals of **8**[OTf] which are isolated.

Yield: 466 mg (91%); **m.p.:** 121 °C; **Raman (100 mW, 298 K, in cm^{-1}):** ν = 3122 (15), 3056 (43), 2964 (57), 2942 (70), 2904 (100), 2783 (9), 1570 (72), 1560 (48), 1464 (26), 1442 (22), 1276 (15), 1223 (15), 1175 (17), 1113 (20), 1044 (61), 1031 (61), 987 (93), 938 (11), 804 (28), 753 (24), 715 (13), 617 (15), 608 (15), 574 (26), 498 (17), 476 (37), 422 (13), 397 (15), 368 (15), 347 (15), 312 (20), 223 (20), 198 (41); **IR (ATR, 298 K, in cm^{-1}):** ν = 3036 (w), 2943 (m), 2927 (m), 2888 (m), 2853 (m), 2709 (w), 1569 (vs), 1556 (s), 1470 (m), 1447 (vs), 1416 (vs), 1388 (m), 1360 (vs), 1268 (m), 1227 (m), 1202 (m), 1167 (s), 1149 (s), 1082 (m), 1046 (m), 1008 (m), 986 (m), 934 (w), 887 (w), 804 (m), 768 (s), 757 (vs), 740 (s), 712 (m), 639 (w), 618 (s), 574 (m), 554 (w), 517 (m), 500 (s), 467 (m), 407 (m); **^{1}H NMR (CD_3CN, 300 K, in ppm):** δ =1.44 (3H, d, $^2J_{HP}$ = 12.95 Hz. C1–H), 1.47-1.50 (9H, m, C10–H), 1.51-1.54 (9H, m, C3–H), 7.39-7.42 (2H, m, C7–H), 7.84-7.91 (4H, m, C5/C6–H), 8.66-8.68 (2H, m, C8–H); **^{13}C{^{1}H} NMR (CD_3CN, 300 K, in ppm):** δ = 5.6-5.8 (1C, m, C1), 25.0-25.1 (3C, m, C3), 28.9 (3C, dt, $^2J_{CP}$ = 15.4 Hz, $^3J_{CP}$ = 5.7 Hz, C10), 32.7-33.2 (1C, m, C9), 36.2-36.6 (1C, m, C2), 122.6 (1C, q, $^1J_{CF3}$ = 321.1 Hz, CF_3), 125.7 (2C, d, $^4J_{CP}$ = 1.8 Hz, C7), 130.5-130.7 (2C, m, C5), 139.1 (2C, m, C6), 152.4-152.5 (2C, m, C8), 155.2-155.6 (2C, m, C4); **^{19}F NMR (CD_3CN, 300 K, in ppm):** δ = –79.3 (3F, s); **^{31}P NMR (CD_3CN, 300 K, in ppm):** A_2MX spin system: δ(P_A) = -70.5 (2P), δ(P_M) = -24.9 (1P), δ(P_X) = 24.2 (1P); $^1J(P_AP_X)$ = -225 Hz, $^1J(P_AP_M)$ = -132

Hz, ^2J(P_MP_X) = 15 Hz; **elemental analysis**: calcd. for $C_{20}H_{29}F_3N_2O_3P_4S$: C: 43.02, H: 5.23, N: 5.02, S: 5.74; found: C: 42.56, H: 4.89, N: 5.04, S: 5.96.

2.9. Preparation of 1,3-bis(1-methylpyridin-1-ium)-4-(*tert*-butyl)-2,2-dimethyltetraphosphetane-2-ium triflate 13[OTf]$_3$

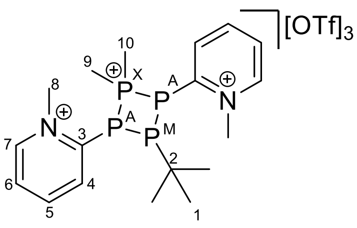

To tetraphosphetane **5** (100 mg, 0.25 mmol) MeOTf (600 µl, 5.5 mmol) is added. After stirring this mixture for 4 h at 80 °C a red solution is obtained. Upon adding Et_2O (2 ml) a colorless precipitate forms which is filtered off and washed with Et_2O (2 x 2 ml) and CH_2Cl_2 (1 x 2 ml). Subsequent evaporation of all volatiles *in vacuo* yields the product as a colorless solid.

Yield: 195 mg (91%); **m.p.:** 212 °C; **Raman (100 mW, 298 K, in cm^{-1}):** v = 3093 (22), 3037 (11), 2963 (52), 2943 (29), 2906 (67), 2864 (18), 1606 (59), 1575 (29), 1491 (19), 1467 (20), 1443 (19), 1400 (15), 1315 (19), 1278 (25), 1227 (25), 1186 (27), 1156 (25), 1091 (22), 1062 (67), 1033 (100), 799 (34), 758 (47), 721 (15), 711 (16), 692 (19), 574 (41), 563 (35), 519 (14), 489 (29), 448 (38), 433 (31), 409 (16), 393 (19), 375 (18), 349 (39), 315 (32), 274 (21), 225 (34), 176 (32); **IR (ATR, 298 K, in cm^{-1}):** v = 3082 (vs), 2986 (vs), 2905 (vs), 1606 (vs), 1575 (vs), 1490 (vs), 1467 (vs), 1445 (vs), 1403 (vs), 1371 (vs), 1248 (vs), 1224 (vs), 1149 (vs), 1090 (vs), 1064 (vs), 1027 (vs), 961 (vs), 912 (vs), 865 (vs), 781 (vs), 758 (vs), 720 (vs), 692 (vs), 635 (vs), 573 (vs), 516 (vs), 499 (vs), 436 (vs), 409 (vs); **^{1}H NMR (CD_3NO_2, 300 K, in ppm):** δ =1.48 (9H, d, $^2J_{HP}$ = 16.64 Hz, C1–H), 2.26 (3H, d, $^2J_{HP}$ = 13.67 Hz, C9/C10–H), 2.83 (3H, dt, $^2J_{HP}$ = 14.31 Hz, $^3J_{HP}$ = 7.10 Hz, C9/C10–H), 4.80 (6H, s, C8–H), 8.27-8.31 (2H, m, C6–H), 8.78-8.83 (2H, m, C5–H), 9.07-9.10 (2H, m, C7–H), 9.10-9.14 (2H, m, C4–H); **^{13}C{^{1}H} NMR (CD_3NO_2, 300 K, in ppm):** δ = 10.2 (1C, d, $^1J_{CP}$ = 21.2 Hz, C9/C10), 17.8 (1C, dtd, $^1J_{CP}$ = 31.3 Hz, $^2J_{CP}$ = 13.5 Hz, $^3J_{CP}$ = 3.3 Hz, C9/C10), 28.9 (3C, dt, $^2J_{CP}$ = 15.0 Hz, $^3J_{CP}$ = 5.0 Hz, C1), 35.8-36.4 (1C, m, C2), 51.4 (2C, *pseudo*-t, J_{CP} = 11.7 Hz, C8), 122.6 (3C, q, $^1J_{CF3}$ = 320.0 Hz, CF_3), 131.6 (2C, s, C6), 139.9 (2C, dd, $^2J_{CP}$ = 22.2 Hz, $^3J_{CP}$ = 4.1 Hz, C4), 147.2 (2C, s, C5), 148.6 (2C, m, C3), 152.7 (2C, s, C7); **^{19}F NMR (CD_3NO_2, 300 K, in ppm):** δ = –79.6 (9F, s); **^{31}P NMR (CD_3NO_2, 300 K, in ppm):** A_2MX spin system: δ(P_A) = -73.5 (2P), δ(P_M) = -12.0 (1P), δ(P_X) = 19.9 (1P); ^1J(P_AP_X) = -228 Hz, ^1J(P_AP_M) = -118 Hz, ^2J(P_MP_X) = 31 Hz; **elemental analysis**: calcd. for $C_{21}H_{29}F_9N_2O_9P_4S_3$: C: 29.87, H: 3.46, N: 3.32, S: 11.39; found: C: 30.31, H: 3.70, N: 3.12, S: 11.57.

2.10. Preparation of 1,2,3-tris(1-methylpyridin-1-ium)-4-(*tert*-butyl)-tetraphosphetane triflate 17[OTf]3

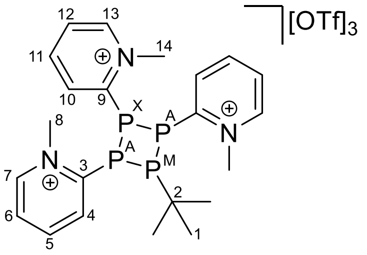

To tetraphosphetanium tristriflate **13**[OTf]3 (109 mg, 0.129 mmol) in 1 ml of MeCN Me_2PPMe_2 (15.8 mg, 0.129 mmol) in 0.2 ml of MeCN is added. The deep red reaction mixture is stirred at r.t. for 4 h. Upon addition of CH_2Cl_2 *via* vapor diffusion at -30 °C colorless crystals of tetraphosphetane **17**[OTf]3 are obtained over the course of 36 h. The mother liquor is decanted of and the crystals are washed with CH_2Cl_2 and are subsequently dried *in vacuo* to yield tetraphosphetane **17**[OTf]3.

Yield: 26 mg (45%); **m.p.:** 275 °C (dec.); **Raman (100 mW, 298 K, in cm^{-1}):** ν = 3091 (22), 2965 (30), 2935 (20), 2897 (24), 2861 (10), 2721 (5), 1610 (47), 1571 (31), 1496 (18), 1442 (17), 1276 (19), 1228 (22), 1181 (46), 1156 (33), 1095 (34), 1068 (80), 1032 (100), 798 (42), 758 (41), 692 (26), 576 (36), 564 (43), 498 (72), 443 (34), 428 (33), 399 (24), 350 (39), 316 (35), 279 (23), 242 (24), 206 (23), 171 (60); **IR (ATR, 298 K, in cm^{-1}):** ν = 3126 (vw), 3089 (vw), 3043 (vw), 2956 (vw), 1608 (vw), 1570 (vw), 1495 (w), 1444 (vw), 1367 (vw), 1313 (vw), 1274 (s), 1245 (vs), 1224 (s), 1176 (m), 1150 (s), 1026 (vs), 896 (vw), 798 (vw), 775 (m), 756 (w), 718 (w), 691 (vw), 635 (vs), 572 (m), 516 (s), 456 (w), 431 (m); **^{1}H NMR (CD_3CN, 300 K, in ppm):** δ =1.41 (9H, d, $^2J_{HP}$ = 15.26 Hz, C1–H), 4.36 (3H, d, $^4J_{HP}$ = 3.23 Hz, C14–H), 4.43 (6H, bs, C8–H), 8.01-8.07 (3H, m, C6/C12–H), 8.56-8.62 (3H, m, C5/C11–H), 8.64-8.68 (1H, m, C10–H), 8.69-8.73 (1H, m, C13–H), 8.75-8.78 (2H, m, C7-H), 8.83-8.87 (2H, m, C4-H); **^{13}C{^{1}H} NMR (CD_3CN, 300 K, in ppm):** δ = 29.1 (3C, dt, $^2J_{CP}$ = 14.7 Hz, $^3J_{CP}$ = 5.2 Hz, C1), 34.8-35.0 (1C, m, C2), 50.2 (2C, bs, C8), 50.4 (1C, d, $^3J_{CP}$ = 11.9 Hz, C14), 122.1 (3C, q, $^1J_{CF3}$ = 322.2 Hz, CF_3), 129.6 (2C, s, C6), 129.8 (1C, s, C12), 138.8-139.2 (1C, m, C10), 139.2-139.6 (2C, m, C4), 145.7 (2C, s, C5), 145.9 (1C, s, C11), 149.9 (1C, s, C13), 149.6 (2C, m, C7), 158.3-158.8 (1C, m, C9), 159.7-160.1 (2C, m, C3); **^{19}F NMR (CD_3CN, 300 K, in ppm):** δ = –79.3 (9F, s); **^{31}P NMR (CD_3CN, 300 K, in ppm):** A_2MX spin system: δ(P_A) = -68.3 (2P), δ(P_M) = -51.1 (1P), δ(P_X) = 2.7 (1P); $^1J(P_AP_X)$ = -123 Hz, $^1J(P_AP_M)$ = -100 Hz, $^2J(P_MP_X)$ = 91 Hz; **elemental analysis**: calcd. for $C_{25}H_{30}F_9N_3O_9P_4S_3$: C: 33.09, H: 3.33, N: 4.63, S: 10.60; found: C: 32.78, H: 3.27, N: 4.61, S: 10.52.

2.11. ^{31}P NMR spectroscopic investigations on the formation of 17^{3+}

To a solution of **13**[OTf]3 (54 mg; 0.064 mmol) in CD_3CN at -30 °C an equimolar amount of Me_2PPMe_2 (7.8 mg; 0.064 mmol; diluted in ca. 100µl MeCN) is added. Keeping the temperature at -30 °C a NMR sample is prepared, which is directly transferred to the pre cooled NMR spectrometer (243 K). ^{1}H-, ^{31}P{^{1}H}- and ^{31}P NMR spectra are measured from 243-293 K in steps of 10 K with a last measurement at 300 K. Additional ^{31}P{^{1}H}^{31}P{^{1}H} COSY experiments have been performed in the beginning at 243 K and in the end at 300 K.

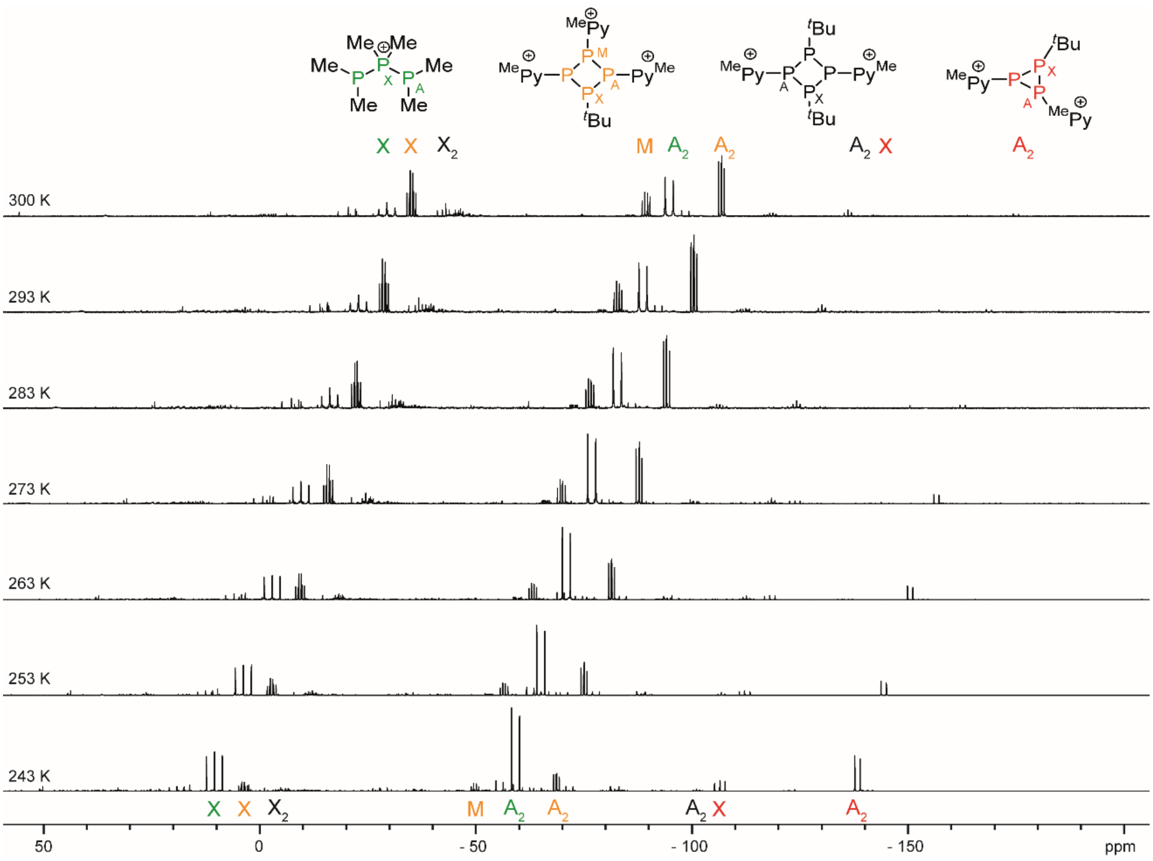

Figure S2.9. ^{31}P{^{1}H} NMR spectra from 243-300 K of the reaction mixture prepared as mentioned above. Identified Resonances are assigned to the respective molecules by their spin systems in the same color as the phosphorus atoms in the molecules shown atop the spectra.

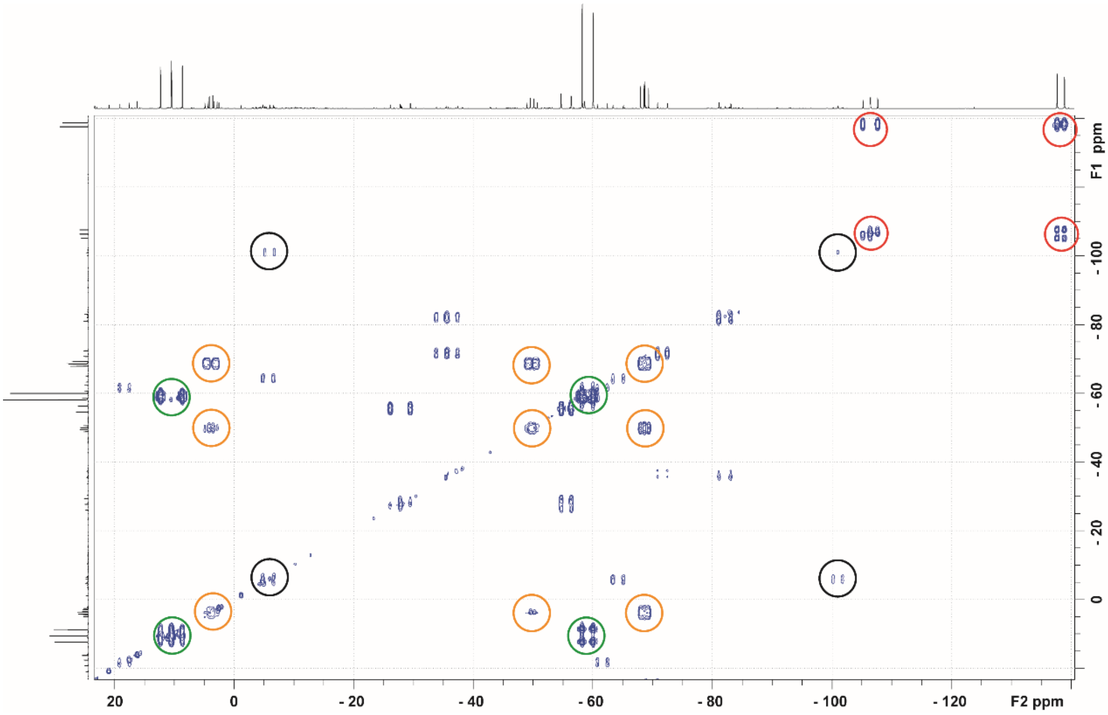

Figure S2.10. 2D NMR spectra from the $^{31}P\{^1H\}^{31}P\{^1H\}$ COSY experiment at 243 K. Cross peaks of identified molecules are highlighted by colored circles, following the color scheme in figure S2.9.

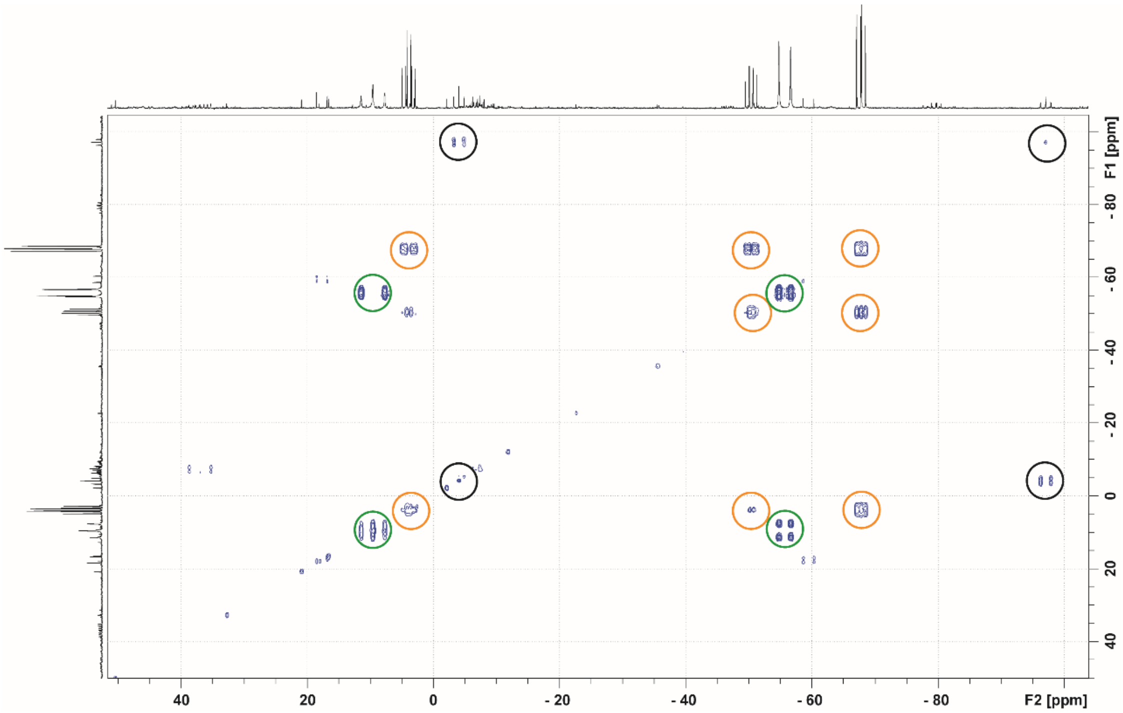

Figure S2.11. 2D NMR spectra from the $^{31}P\{^1H\}^{31}P\{^1H\}$ COSY experiment at 300 K. Cross peaks of identified molecules are highlighted by colored circles, following the color scheme in figure S2.9.

3. X-ray Diffraction Refinements

Suitable single crystals were coated with Paratone-N oil or Fomblin Y25 PFPE oil, mounted using either a glass fiber or a nylon loop and frozen in the cold nitrogen stream. Crystals were measured at low temperature on a Rigaku Oxford Diffraction SuperNova diffractometer using a Cu micro focus X-ray source. Crystal and data collection details are given in Tables **S3.1** to **S3.4**. Data reduction and absorption correction was performed either with CrysaAlisPro[5] software. Using Olex2[6], the structures were solved with SHELXS/T[7] by direct methods and refined with SHELXL[8] by least-square minimization against F^2 using first isotropic and later anisotropic thermal parameters for all non-hydrogen atoms. Hydrogen atoms bonded to carbon atoms were added to the structure models on calculated positions using the riding model. All other hydrogen atoms were localized in the difference Fourier map. Images of the structures were produced with Olex2[6] software.

The X-ray crystallographic coordinates for structures reported in this article have been deposited at the Cambridge Crystallographic Data Centre (CCDC), under CCDC deposition numbers 1990335-1990345. These data can be obtained free of charge from the CCDC via http://www.ccdc.cam.ac.uk/data_request/cif.

Table 3.1: Crystallographic data and details of the structure refinements of **4** and **5**.

	**4**	**5**
formula	$C_{20}H_{28}P_4$	$C_{18}H_{26}N_2P_4$
M_r in g mol^{-1}	392.30	394.29
color, habit	Colorless, block	Clear, colorless, block
crystal system	Triclinic	Triclinic
space group	*P*-1	*P*-1
a in Å	9.1061(3)	8.32598(17)
b in Å	10.8015(4)	10.35329(18)
c in Å	11.0270(4)	13.62138(17)
α in °	75.983(3)	107.2865(13)
β in °	89.224(3)	90.3167(14)
γ in °	85.882(3)	109.4435(17)
V in Å^3	1049.40(7)	1050.07(3)
Z	2	2
T in K	100.01(10)	100.01(10)
crystal size in mm^3	0.204 x 0.109 x 0.08	0.166 × 0.116 × 0.077
ρ_c in g cm^{-3}	1.242	1.247
F(000)	416.0	416.0
diffractometer	Super Nova	Super Nova
$\lambda_{XK\alpha}$ in Å	X = Cu<br>1.54184	X = Cu<br>1.54184
θ_{min} in °<br>θ_{max} in °	8.266<br>153.07	6.842<br>153.072
index range	$-11 \le h \le 11$<br>$-9 \le k \le 13$<br>$-13 \le l \le 13$	$-10 \le h \le 10$<br>$-12 \le k \le 12$<br>$-16 \le l \le 17$
μ in mm^{-1}	3.304	3.333
abs. correction	Multi-Scan	Gaussian
reflections collected	9775	9375
reflections unique	4351	4346
R_{int}	0.0185	0.0196
reflections obs. [F>2σ(F)]	4156	4141
residual density in e Å^{-3}	0.30, –0.27	0.35, –0.30
parameters	223	240
GOOF	1.026	1.033
R_1 [I>2σ(I)]	0.0244	0.0293
wR_2 (all data)	0.0645	0.0789
CCDC	1990335	1990345

Table 3.1: Crystallographic data and details of the structure refinements of [Cu**5**$_2$][OTf]*CH_2Cl_2, [Ag**5**$_2$][OTf]*CH_3CN and [Au**5**$_2$][OTf]*CH_2Cl_2.

	[Cu**5**$_2$][OTf]*CH_2Cl_2	[Ag**5**$_2$][OTf]*CH_3CN	[Au**5**$_2$][OTf]*CH_2Cl_2
formula	$C_{38}H_{54}Cl_2CuF_3N_4O_3P_8S$	$C_{39}H_{55}AgF_3N_5O_3P_8S$	$C_{38}H_{54}AuCl_2F_3N_4O_3P_8S$
M_r in g mol^{-1}	1086.11	1086.57	1219.54
color, habit	Clear yellow, block	Clear colorless, block	Clear colorless, block
crystal system	Triclinic	Triclinic	Triclinic
space group	*P*-1	*P*-1	*P*-1
a in Å	14.9813(3)	12.6802(3)	14.8924(2)
b in Å	16.6700(3)	14.2616(4)	16.7756(2)
c in Å	22.63748(19)	16.8114(4)	22.8908(4)
α in °	72.9307(11)	65.300(3)	73.4752(13)
β in °	82.9453(11)	89.294(2)	83.6786(13)
γ in °	67.1309(17)	67.278(3)	67.3194(13)
V in $Å^3$	4979.38(15)	2507.03(13)	5058.59(14)
Z	4	2	4
T in K	100.01(10)	100.0(3)	100.01(10)
crystal size in mm^3	0.264 x 0.142 x 0.064	0.257 x 0.156 x 0.138	0.178 x 0.087 x 0.041
ρ_c in g cm^{-3}	1.449	1.439	1.601
F(000)	240.0	1116.0	2440.0
diffractometer	Super Nova	Super Nova	Super Nova
$\lambda_{XK\alpha}$ in Å	X = Cu<br>1.54184	X = Cu<br>1.54184	X = Cu<br>1.54184
θ_{min} in °<br>θ_{max} in °	5.974<br>153.786	5.88<br>153.426	5.92<br>153.442
index range	$-16 \le h \le 18$<br>$-21 \le k \le 20$<br>$-28 \le l \le 26$	$-15 \le h \le 15$<br>$-16 \le k \le 17$<br>$-21 \le l \le 20$	$-17 \le h \le 18$<br>$-20 \le k \le 21$<br>$-28 \le l \le 28$
μ in mm^{-1}	4.861	6.464	9.642
abs. correction	Gaussian	Gaussian	Gaussian
reflections collected	50722	25869	72942
reflections unique	20368	10409	21100
R_{int}	0.0416	0.0206	0.0214
reflections obs. [F>2σ(F)]	18310	10196	20520
residual density in e $Å^{-3}$	1.05, –0.86	1.50, –1.31	1.28, –1.78
parameters	1105	568	1105
GOOF	1.044	1.024	1.038
R_1 [I>2σ(I)]	0.0416	0.0265	0.0234
wR_2 (all data)	0.1191	0.0670	0.0584
CCDC	1990336	1990340	1990341

Table 3.2: Crystallographic data and details of the structure refinements of **6**[OTf], **8**[OTf], and **11**[OTf]$_2$.

	**6**[OTf]	**8**[OTf]	**11**[OTf]$_2$
formula	$C_{22}H_{31}F_3O_3P_4S$	$C_{20}H_{29}F_3N_2O_3P_4S$	$C_{22}H_{32}F_6N_2O_6P_4S_2$
M_r in g mol^{-1}	556.41	558.39	722.49
color, habit	Clear, colorless, block	Clear, colorless, block	Colorless, block
crystal system	Orthorhombic	Monoclinic	Orthorhombic
space group	$P2_12_12_1$	$P2_1/c$	*Pccn*
a in Å	10.94462(9)	13.97178(9)	11.21381(12)
b in Å	14.08128(12)	15.50651(11)	19.60180(19)
c in Å	17.64605(14)	24.38356(18)	13.59001(18)
α in °	90	90	90
β in °	90	94.7277(6)	90
γ in °	90	90	90
V in Å^3	2719.51(4)	5264.81(6)	2987.23(6)
Z	4	8	4
T in K	100.01(10)	100.01(10)	99.9(5)
crystal size in mm^3	0.259 x 0.169 x 0.092	0.192 × 0.099 × 0.084	0.216 × 0.076 × 0.053
ρ_c in g cm^{-3}	1.359	1.409	1.606
F(000)	1160.0	2320.0	1488.0
diffractometer	Super Nova	Super Nova	Super Nova
$\lambda_{XK\alpha}$ in Å	X = Cu<br>1.54184	X = Cu<br>1.54184	X = Cu<br>1.54184
θ_{min} in °<br>θ_{max} in °	8.032<br>153.262	6.348<br>153.41	9.022<br>153.29
index range	$-7 \leq h \leq 13$<br>$-16 \leq k \leq 17$<br>$-22 \leq l \leq 18$	$-17 \leq h \leq 17$<br>$-19 \leq k \leq 17$<br>$-27 \leq l \leq 30$	$-14 \leq h \leq 13$<br>$-15 \leq k \leq 24$<br>$-17 \leq l \leq 16$
μ in mm^{-1}	3.662	3.808	4.367
abs. correction	Gaussian	Gaussian	Gaussian
reflections collected	15014	50820	14708
reflections unique	5654	11024	3118
R_{int}	0.0197	0.0229	0.0213
reflections obs. [F>2σ(F)]	5186	10434	2536
residual density in e Å^{-3}	0.17, –0.24	0.86, –0.48	0.61, –0.55
parameters	305	628	194
GOOF	1.043	1.032	1.064
R_1 [I>2σ(I)]	0.0190	0.0343	0.0330
wR_2 (all data)	0.0481	0.0895	0.0889
CCDC	1990339	1990337	1990344

Table 3.3: Crystallographic data and details of the structure refinements of **12**[OTf]$_3$*2 CH_3NO_2, **13**[OTf]$_3$*CH_3NO_2 and **17**[OTf]$_3$.

	**12**[OTf]$_3$*2 CH_3NO_2	**13**[OTf]$_3$*CH_3NO_2	**17**[OTf]$_3$
formula	$C_{26}H_{41}F_9N_4O_{13}P_4S_3$	$C_{22}H_{32}F_9N_3O_{11}P_4S_3$	$C_{25}H_{30}F_9N_3O_9P_4S_3$
M_r in g mol^{-1}	1008.69	905.56	907.58
color, habit	Clear, colorless, block	Clear colourless, block	Clear yellow, block
crystal system	Triclinic	Triclinic	Monoclinic
space group	*P*-1	*P*-1	$P2_1/c$
a in Å	13.3125(3)	13.1277(3)	11.97360(10)
b in Å	13.4806(3)	13.5583(3)	12.42100(10)
c in Å	13.9690(4)	22.2736(4)	24.8508(2)
α in °	61.919(2)	98.981(2)	90
β in °	84.582(2)	97.504(2)	94.1930(10)
γ in °	71.999(2)	104.540(2)	90
V in Å^3	2099.62(10)	3730.20(14)	3686.02(5)
Z	2	4	4
T in K	100.00(10)	100.00(10)	100.00(10)
crystal size in mm^3	0.221 × 0.198 × 0.078	0.593 × 0.316 × 0.186	0.347 × 0.233 × 0.169
ρ_c in g cm^{-3}	1.595	1.612	1.635
F(000)	1036.0	1848.0	1848.0
diffractometer	Super Nova	Super Nova	Super Nova
$\lambda_{XK\alpha}$ in Å	X = Cu<br>1.54184	X = Cu<br>1.54184	X = Cu<br>1.54184
θ_{min} in °<br>θ_{max} in °	6.994<br>153.582	6.87<br>153.692	7.134<br>153.822
index range	$-14 \leq h \leq 16$<br>$-17 \leq k \leq 16$<br>$-17 \leq l \leq 17$	$-16 \leq h \leq 14$<br>$-15 \leq k \leq 16$<br>$-25 \leq l \leq 27$	$-15 \leq h \leq 14$<br>$-12 \leq k \leq 15$<br>$-31 \leq l \leq 31$
μ in mm^{-1}	3.993	4.372	4.388
abs. correction	Gaussian	Gaussian	Gaussian
reflections collected	23898	41107	39477
reflections unique	8722	15525	7710
R_{int}	0.0158	0.0415	0.0369
reflections obs. [F>2σ(F)]	8518	14705	6974
residual density in e Å^{-3}	0.43, –0.57	0.65, –0.91	0.40, –0.38
parameters	543	1171	690
GOOF	1.030	1.149	1.047
R_1 [I>2σ(I)]	0.0288	0.0741	0.0362
wR_2 (all data)	0.0766	0.1533	0.0968
CCDC	1990342	1990338	1990343

4. References

[1] R. Schoemaker, K. Schwedtmann, A. Franconetti, A. Frontera, F. Hennersdorf, J. J. Weigand, *Chem. Sci.* **2019**, *10*, 11054.

[2] G. Becker, O. Mundt, M. Rössler, E. Schneider, *Z. anorg. allg. Chem.* **1978**, *443*, 42.

[3] B. A. Pindzola, J. Jin, D. L. Gin, *J. Am. Chem. Soc.* **2003**, *125*, 2940.

[4] S. Ahrland, K. Dreisch, B. Norén, A. Oskarsson, *Materials Chemistry and Physics* **1993**, 35, 281-289

[5] CrysAlisPRO, Oxford Diffraction /Agilent Technologies UK Ltd, Yarnton, England.

[6] O. V. Dolomanov, L. J. Bourhis, R. J. Gildea, J. A. K. Howard, H. Puschmann, *J. Appl. Cryst.* **2009**, *42*, 339-341.

[7] G. Sheldrick, *Acta Cryst.* **2008**, *64*, 112-122.

[8] G. Sheldrick, *Acta Cryst. C* **2015**, *71*, 3-8.
